# In situ spectroscopy-guided engineering of rhodium single-atom catalysts for CO oxidation

**DOI:** 10.1038/s41467-019-09188-9

**Published:** 2019-03-22

**Authors:** Max J. Hülsey, Bin Zhang, Zhirui Ma, Hiroyuki Asakura, David A. Do, Wei Chen, Tsunehiro Tanaka, Peng Zhang, Zili Wu, Ning Yan

**Affiliations:** 1 Department of Chemical and Biomolecular Engineering, National University of Singapore, 4 Engineering Drive 4, 117585 Singapore, Singapore; 2 Department of Chemistry, National University of Singapore, 3 Science Drive 3, 117543 Singapore, Singapore; 3 Department of Molecular Engineering, Graduate School of Engineering, Kyoto University, Kyoto, 615-8510 Japan; 4 Elements Strategy Initiative for Catalysts & Batteries (ESICB), Kyoto University, Kyoto, 615-8245 Japan; 5 Department of Chemistry, Dalhousie University, Halifax, NS B3H 4R2 Canada; 6 Department of Physics, National University of Singapore, 2 Science Drive 3, 117542 Singapore, Singapore; 7 Chemical Sciences Division, Oak Ridge National Laboratory, Oak Ridge, Tennessee 37831-6133 United States

## Abstract

Single-atom catalysts have recently been applied in many applications such as CO oxidation. Experimental in situ investigations into this reaction, however, are limited. Hereby, we present a suite of operando/in situ spectroscopic experiments for structurally well-defined atomically dispersed Rh on phosphotungstic acid during CO oxidation. The identification of several key intermediates and the steady-state catalyst structure indicate that the reactions follow an unconventional Mars-van Krevelen mechanism and that the activation of O_2_ is rate-limiting. In situ XPS confirms the contribution of the heteropoly acid support while in situ DRIFT spectroscopy consolidates the oxidation state and CO adsorption of Rh. As such, direct observation of three key components, i.e., metal center, support and substrate, is achieved, providing a clearer picture on CO oxidation on atomically dispersed Rh sites. The obtained information are used to engineer structurally similar catalysts that exhibit T_20_ values up to 130 °C below the previously reported Rh_1_/NPTA.

## Abstract (web summary)

Single-atom catalysts have been studied for CO oxidation, but experimental in situ investigations are limited. Here, the authors use a suite of in situ/operando spectroscopy to identify key intermediates and define design principles to enhance the CO oxidation activity of atomically dispersed Rh on heteropoly acids.

## Introduction

Single-atom catalysts (SACs), where metal atoms are atomically dispersed on the support, have emerged as a new frontier in catalysis in recent years^1–8^. Many exciting systems were developed, often based on noble metals such as Pt^9–18^, Pd^19–23^, Rh^24–31^, and Au^32,33^ but also on earlier transition metals^34–39^ with a number of catalytic applications. However, due to the complex surface structure of most metal oxides and other types of supports, the exact coordination environments of the supported isolated atoms are widely unknown thus hampering mechanistic understanding both experimentally and theoretically.

CO oxidation is among the earliest^40^ and the most frequently investigated^9,12,16,26,40–47^ reactions for SACs. Mechanistic studies on single-atom catalytic systems for CO oxidation, however, are under intensive debate in the literature^9,41^. Conventional Langmuir-Hinshelwood (L–H), Eley-Rideal (E–R), and Mars-van Krevelen (M–vK) mechanisms are all discussed. A combination of both L–H and E–R mechanisms is also proposed where the first molecule of CO is oxidized in an L–H manner via the formation of a peroxide-like intermediate followed by the oxidation of another CO molecule with the remaining oxygen on the single atom^48^. Another variation of the E–R mechanism suggests the O_2_ activation promoted by CO on silver SACs including the simultaneous oxidation of two adsorbed CO molecules by one O_2_ molecule from the gas phase^49^. Atomically dispersed Pt supported on CeO_2_ were shown to follow a water-mediated M–vK pathway for the oxidation of CO via the formation of a carboxyl-intermediate which can be rapidly dehydrogenated by surface hydroxyl groups^43^. Although thorough kinetic studies on the single-atom catalyzed CO oxidation are rare, reaction orders towards O_2_ are normally proposed to be in the range of 0–0.5 depending on the reaction pathway but have been reported to be 1 in our previous study which hints at an unconventional CO oxidation mechanism on SACs. Those previous investigations leave ambiguity about the exact reaction mechanisms for different SAC systems with some even claiming that SACs exhibit no reactivity in CO oxidation and the water–gas shift reaction^42^.

The controversial mechanisms proposed in the literature highlight the importance of performing in situ spectroscopic investigation on structurally simple and well-defined SACs to understand the roles of metal, support, and the fate of CO during a full catalytic cycle. Although in situ spectroscopic experiments have proven success in elucidating the structure and active sites of SACs under reaction conditions^50,51^, for CO oxidation it has so far been primarily limited to IR^42,45^ and XPS^46^ spectroscopy, tools that only directly or indirectly provide information on the oxidation state of the catalyst. Operando or in situ spectroscopic techniques that provide direct information on both the oxidation state and coordination environment of isolated atoms, such as X-ray absorption spectroscopy (XAS), have not yet been employed, let alone the combined use of operando XAS with other in situ techniques. Furthermore, the rational design of SACs with improved catalytic performance based on mechanistic understanding is lacking so far.

A major disadvantage of in situ spectroscopy study is that for spectra measured at an ensemble level an averaged signal of all responsive species is collected, which may hide the information from the real active species and thus be misleading. Only when all species are identical will the spectra reflect accurate information of individual species, and only then the spectra could be conveniently used to reveal structural information and reaction mechanism. We recently reported a SAC system supported on heteropoly acids (HPA)^44,52^, where the coordination environment of each individual atom is clearly defined and investigated by XAS and DFT calculations rendering this catalyst ideal for in-depth in situ spectroscopic investigations. An additional benefit of using HPAs is the tunability of the support towards lower or higher redox potential by simply varying the molecular composition of the HPA.

**Fig. 1:**
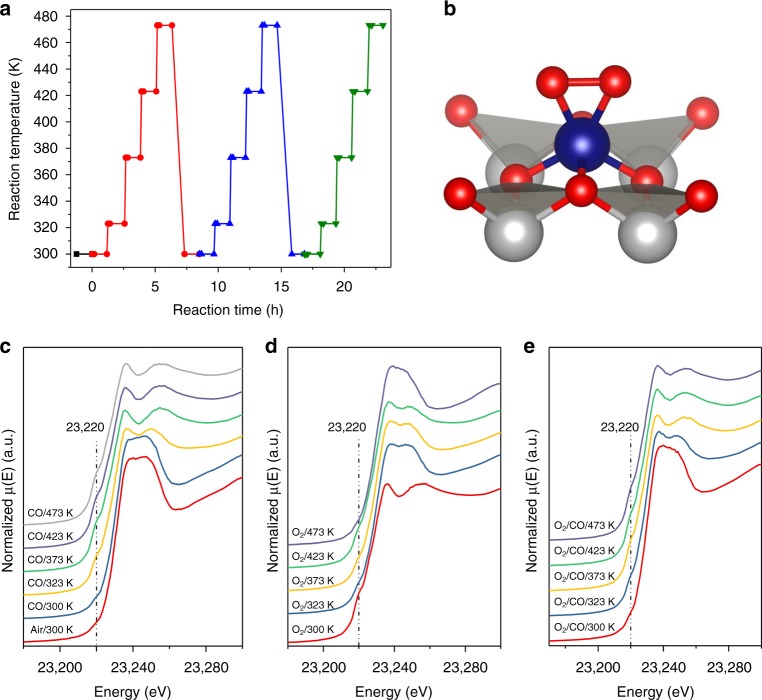
Course of the operando experiments and correlating operando spectra. **a** Operando XAS experiments at the Rh K-edge under different atmospheres and reaction temperatures: (black squares) air; (red circles) 5% CO/He; (blue triangles) 7% O_2_/He; (green down-pointing triangles) 2.9% CO/2.9% O_2_/He. **b** Proposed catalyst structure before exposure to CO with one oxygen molecule bound to Rh. Atom colors: W (gray), O (red), C (black), Rh (blue). Rh K-edge spectra under **c** CO, **d** O_2_, and **e** O_2_/CO at increasing temperatures (from bottom to top). The dashed lines are highlighting the pre-edge feature and have no physical meaning

Here, we present operando XAS experiments, corroborated with in situ DRIFTS and XPS analysis, to investigate the active site of a noble metal single-atom catalyst in CO oxidation, based on a Rh-HPA catalyst that exhibits an onset temperature of 423 K. Extended X-ray absorption fine structure (EXAFS) and X-ray absorption near edge structure (XANES) measurements on the Rh K-edge are taken in regular periods under air, dilute CO, dilute O_2_, and then a mixture of CO and O_2_ at different temperatures below and above the onset temperature but in a kinetically limited regime (Fig. 1a and Supplementary Figure 1) to reveal the electronic state and coordination environment of the catalyst under reaction conditions. The production of carbon dioxide is monitored online by a mass spectrometer (Supplementary Figure 2). A similar procedure is used for experiments for the operando/in situ W L_III_-edge (Supplementary Figure 3) XAS, XPS, and DRIFTS studies. Subsequently, three additional HPA-supported rhodium SACs are synthesized, characterized, and tested in the CO oxidation reaction whose eactivity behavior match the predictions by mechanistic understanding.

## Results

### Synthesis and characterization of Rh_1_/NPTA

The catalyst synthesis relies on the strong electrostatic adsorption of a rhodium cation on the negatively charged heteropoly acid molecules. To achieve the immobilization of one rhodium atom on a heteropoly acid molecule, a stoichiometric ratio of around 1:4 was chosen for the synthesis. Upon precipitation of the soluble rhodium-HPA adduct, a stable, self-supported SAC (Rh_1_/NPTA) can be obtained exhibiting good activity in the oxidation of CO. The particle size is around 200 nm (Supplementary Figure 4) with homogeneously dispersed oxygen, tungsten, rhodium and phosphorus (Supplementary Figure 5). As shown before^52^, heteropoly acids offer a very limited amount of binding sides due to their high symmetry and all rhodium atoms are expected to be located at the same 4-fold hollow site due to its high stability. No Rh–Rh scattering for the Rh_1_/NPTA catalyst is observed and the white line intensity is similar to the Rh_2_O_3_ reference sample in the Rh K-edge EXAFS and XANES spectra (Supplementary Figures 6a and 6b). The first indicates the dispersion of single Rh atoms whereas the latter relates to positively charged Rh species (3+) at the rest state. Furthermore, the W L_III_-edge XANES and EXAFS spectra reveal the stability of the phosphotungstic acid after adsorption of rhodium (Supplementary Figures 7a and 7b). It is expected that the initial catalyst bears a structure as shown in Fig. 1b, where one oxygen molecule is bound to the rhodium single atoms, based on XAFS fitting results^44^.

### Oxygen vacancy formation during reductive catalyst treatment

The catalyst was first treated in CO flow with temperature stepwise-increased from 300 K to 473 K. The most striking features in the XANES spectra of the Rh SAC after the reductive treatment is the occurrence of two intensive peaks at around 23237 and 23255 eV indicating changes in the structure of the catalyst. A bit less obvious is the formation of a pre-edge feature at around 23220 eV (Fig. 1c and Supplementary Figure 8c). Both changes start to appear at a temperature as low as 323 K, and finish at 373 K, well below the onset temperature of 423 K. Although the appearance of pre-edge features is not often reported for 4d and 5d metals, a similar pattern has been observed earlier for Ru-based catalysts. Upon distortion of the almost octahedral symmetry at the metal center, the 5p and 4d orbitals are mixing more strongly thus enhancing the intensity of electric dipole forbidden transitions from 1s to 4d orbitals^53–55^.

**Fig. 2:**
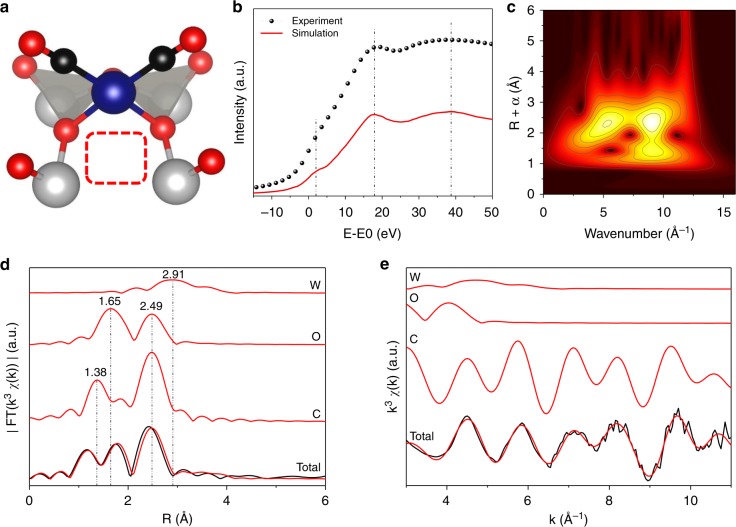
Spectroscopic evidence for the formation of an oxygen vacancy. **a** DFT-optimized structure of the catalyst with oxygen vacancy and two bound CO molecules. Atom colors: W (gray), O (red), C (black), Rh (blue). **b** best fit for the XANES simulation of the catalyst structure depicted in **a**; **c** Morlet wavelet transform of the catalyst after treatment under CO. **d**, **e** EXAFS analysis of structure (**a**) in **d** R and **e** k space. Curves from bottom to top are the experimental scattering contributions (black) superimposed with the total fitted scattering contributions, Rh–C, Rh–O, and Rh–W scatterings (all in red)

In the context of CO oxidation on a reducible support, this indicates the loss of one oxygen atom in the heteropoly acid support. Subsequently, ab initio XANES prediction on several tetra-, penta-, or hexa-coordinated DFT-optimized structural models were conducted. This method is known to not reproduce intensities very well^56^ but all three key features including the pre-edge region of the XANES spectrum are predicted at their exact location for the penta-coordinated structure with the oxygen vacancy and two adsorbed CO molecules (Fig. 1d, e and Supplementary Figure 8d). A simulation for the hexa-coordinated structure without oxygen vacancy and two CO molecules neither yielded a reasonable fit for the pre-edge nor represented the white line features (Supplementary Figure 9a). Although the pre-edge feature is reasonably well captured for the penta-coordinated structure with oxygen vacancy and one bound CO molecule, the other peaks do not match (Supplementary Figure 9b). After comparison, the structure with two bound CO molecules and the oxygen vacancy (Fig. 2a) was found to give the best alignment between experiment and theory (Fig. 2b). An analysis of the EXAFS scattering contributions revealed a decrease in the first shell and a pronounced increase in the second shell. This can be assigned to scattering contributions from oxygen and carbon monoxide, respectively. EXAFS fitting gives a good overlap between the DFT-optimized structure and the experimental spectra up to a range of around 3 Å around the scattering atoms (Fig. 2d, e). Morlet wavelet analysis of the spectrum after reduction under CO verified (Fig. 2c) that there was no scattering contribution from rhodium particle or cluster formation. Although the wavenumber for the first shell radial distance is higher (9.0 Å^−1^) than the 7.3 Å^−1^ estimated for Rh_2_O_3_ (Supplementary Figure 10c) it is still significantly below 10.6 Å^−1^ as estimated for rhodium foil (Supplementary Figure 10d). The contribution at 1.2 Å radial distance plausibly comes from a light element such as oxygen. The occurrence of a scattering partner at even lower wavenumber for higher radial distances hint at the adsorption of carbon as a lighter scattering atom similar to a study reported earlier^57^ and thus further corroborates the existence of a CO-bound structure (as shown in Fig. 2a).

Along the CO treating process, the white line intensity of Rh is reduced at merely 323 K and drops entirely at 373 K (Fig. 1c and Supplementary Figure 8a). Meanwhile, CO_2_ was detected by the online mass spectrometer as soon as CO was switched on (Supplementary Figure 2). The trend of the white line intensity change matches well with the intensity change of the pre-edge features. This, together with the fact that CO_2_ was observed at ambient temperature (without O_2_), highlight the reduction of Rh^3+^ to Rh^+^ readily occurs on Rh_1_/NPTA, which is associated with an oxygen vacancy creation in the support via oxygen transfer from PTA to CO forming CO_2_ (Fig. 1c). Treatment of the catalyst under CO correlates to Eq. 1, the conversion of one CO into one CO_2_ molecule by the support leaving behind an oxygen vacancy (indicated by the dashed circle). The subsequent adsorption of another CO molecule on rhodium can be expected to be very fast as the calculated adsorption energy of two CO molecules is high^58^.1

### Re-oxidation of the catalyst under oxygen

**Fig. 3:**
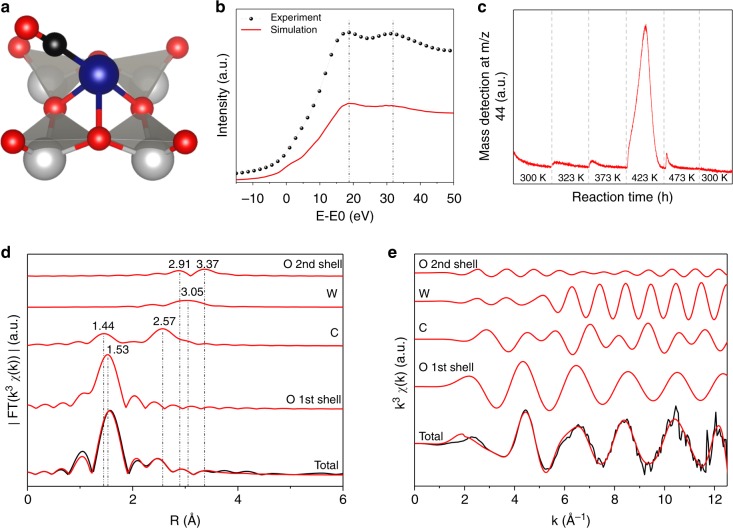
Re-oxidation of the catalyst under oxygen atmosphere. **a** DFT-optimized structure of the intermediate of the catalyst under oxidative conditions. Atom colors: W (gray), O (red), C (black), Rh (blue). **b** best fit for the XANES simulation of the catalyst structure depicted in **a**. **c** Temperature dependence of the formation of CO_2_ during the treatment under O_2_ as determined by online mass spectrometry. **d**, **e** EXAFS analysis of structure **a** in **d** R and **e** k space. Curves from bottom to top are the experimental scattering contributions (black) superimposed with the total fitted scattering contributions, Rh–C, Rh–O (1^st^ and 2^nd^ shell) and Rh–W scatterings (all in red)

Re-oxidation of Rh by O_2_ to an intermediate level occurs at temperatures as low as 323 K (Fig. 1d and Supplementary Figure 8b). However, at 423 K the white line intensity drops again indicating a decrease in oxidation state. This correlates well with the formation of CO_2_ at the same temperature as shown by mass spectroscopy (Fig. 3c). A comparison of the experimental XANES spectrum at 423 K with the simulated XANES spectra (Fig. 3b) reveals a significant overlap with the penta-coordinated structure without oxygen vacancy and with a single bound CO molecule (Fig. 3a). As indicated earlier, the occurrence of the pre-edge feature hints at the loss of centrosymmetry around rhodium, in this case by the removal of one CO molecule. At 473 K, the centrosymmetry is regained by the removal of carbon monoxide in accordance with CO DRIFT spectroscopy under O_2_ (Supplementary Figure 12). When the release of CO_2_ is complete, the white line intensity increases again to its maximum indicating that Rh is in the 3^+^ state again (Fig. 1d and Supplementary Figure 8b). The intensity of the pre-edge peak drops significantly at low temperatures (323 and 373 K) but increases again during the formation of CO_2_ upon which the oxygen vacancy seems to be refilled completely (Fig. 1d and Supplementary Figure 8d and Eq. 2 and 3). EXAFS fitting of the structure observed at 423 K under O_2_ reveals scattering contributions mainly from the first shell from oxygen but also from higher shell carbon, tungsten, and oxygen scattering (Fig. 3d, e).23

A very similar procedure was chosen for operando XAS experiments at the W L_III_-edge (Supplementary Figure 3b) but with a low Rh to W molar ratio, little changes in the XANES and EXAFS are to be expected. Indeed, the white line intensity only changes marginally during the course of the reaction (Supplementary Figures 7c and 7d) and certain changes in the EXAFS spectra (Supplementary Figure 11a) are probably caused by thermal effects^59^.

Comparing the temperatures at which the catalyst is reduced under CO and re-oxidized under O_2_ provides insights into the relative rates of individual steps in a catalytic cycle. When exposed to CO, Rh is partially reduced even at 323 K and fully reduced at 373 K. In accordance with that is the oxygen vacancy creation on the support and CO_2_ formation in the gas phase. In contrast, the re-oxidation of Rh completes at a much higher temperature of 473 K, while the formation of CO_2_ under oxygen atmosphere is observed only when temperature increases to 423 K, matching very well with the onset temperature of the catalyst. Based on these, the Rh SAC follows an unconventional M–vK mechanism where the re-oxidation of metal center and the re-generation of the oxygen site on the support seems to be the rate-controlling step. In an atmosphere containing CO, the adsorption of another molecule of CO can again be expected to occur rapidly regenerating the catalyst (Eq. 4).4

### Investigation of the catalyst under steady-state

In addition to the analysis of the catalyst under pure O_2_ and CO atmospheres, it is also monitored under a mixture of O_2_ and CO at various temperatures. The white line intensity gradually drops and reaches its minimum at 373 K (Fig. 1e). In the meantime, the pre-edge feature intensity increases to the same value as observed after treatment of the catalyst under CO. This indicates that the steady-state of the catalyst contains a Rh^+^ species and has the same oxygen vacancy as after reduction with CO. Even after the onset of the CO oxidation (Supplementary Figure 2) at 423 K, those features prevail. These observations are exactly to be expected if the refilling of oxygen vacancy is the rate-limiting step, and are in line with the fact that the reaction has a first order dependence on O_2_ and zeroth order dependence on CO as shown earlier^44^.

### In situ XPS

**Fig. 4:**
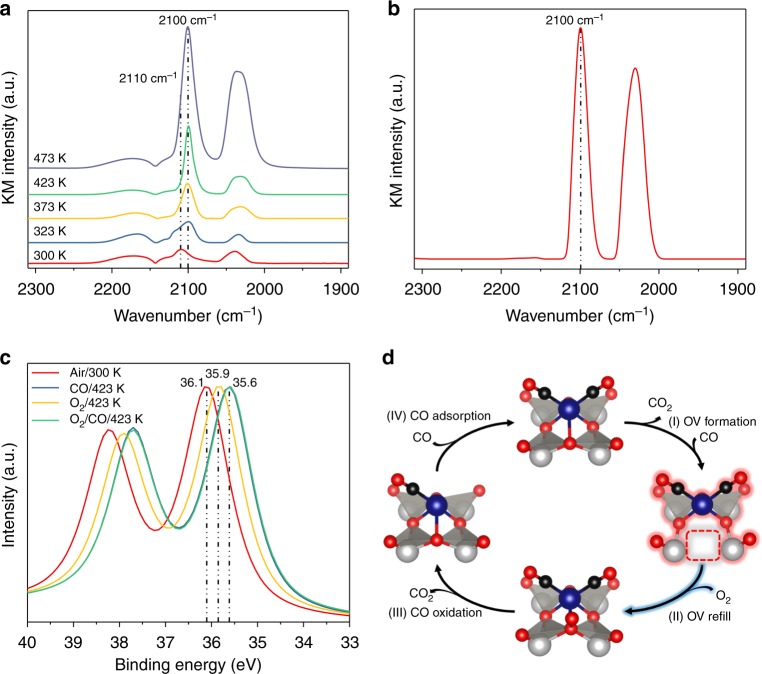
In situ DRIFT and XPS spectroscopy and proposed reaction cycle. CO DRIFT spectra of **a** the catalyst under CO at increasing temperatures (from bottom to top) and **b** of the catalyst under CO/O_2_ at 473 K. **c** In situ W 4*f*_7/2_ XPS spectra of the catalyst under different gas atmospheres and temperatures. **d** Key steps of the proposed reaction mechanism based on in situ spectroscopy; the steady-state catalyst structure and the corresponding rate-controlling step are highlighted. Atom colors: W (gray), O (red), C (black), Rh (blue); OV stands for oxygen vacancy

Because the observed changes in the oxidation state of tungsten were small according to operando XAS, XPS was chosen as a complimentary technique to investigate the oxidation state of tungsten during the CO oxidation. The binding energy of the W 4*f*_7/2_ peak shifted from 36.1 to 35.6 eV (Fig. 4c) indicating the reduction of W in the support to occur at close to room temperature. The reduction finishes at 423 K, (Supplementary Figure 13) higher than the full reduction temperature of Rh in the operando XAS study (373 K), presumably due to the lower CO pressure applied in XPS analysis, or the different catalyst pellet preparation procedures. Those results indicate that the support is actively contributing to the oxidation of CO and is reduced. The rather small changes in the XPS spectra and the missing formation of separate peaks can be explained by the charge delocalization over the heteropoly acid support^60^. As reported earlier, a change in the tungsten oxidation state between 6^+^ and 5^+^ can be indicated by a change in the binding energy of as large as 3 eV (a more detailed analysis is provided in Supplementary Table 2) explaining the average change of ~0.5 eV. Under O_2_ atmosphere, the tungsten species are gradually re-oxidized until 423 K (Supplementary Figure 14). As observed by operando XAS, the catalyst does not reach the initial oxidation state before temperature reaches 473 K. Exposing the catalyst to a mixture of O_2_ and CO leads to an immediate reduction of the tungsten species to binding energies of 35.6 eV (Supplementary Figure 15) further confirming the existence of the oxygen vacancy during reaction conditions.

### In situ DRIFT spectroscopy

For further consolidation of the adsorbates and the oxidation state changes during the treatment of the catalyst under reaction conditions, in situ DRIFTS experiments were conducted similar to the operando XAS experiments. CO is a commonly used gas to monitor the dispersion and oxidation state of SACs^45^. Typically, a splitting of the CO vibration on *gem*-dicarbonyl Rh species is observed, which is assigned to the symmetric and asymmetric vibration of two CO molecules^27^. In the initial state, two CO frequencies were observed at 2110 and 2039 cm^−1^, respectively (Fig. 4a), assignable to Rh(CO)_2_^3+^ species^25^. Upon exposure to CO under increasing temperatures, a redshift of the band from 2110 to 2100 cm^−1^ was observed starting at 323 K, indicating the formation of Rh(CO)_2_^+^ species. This is in good alignment with the operando XANES experiments. The same trend is confirmed by ab initio simulations of CO vibrations. Upon formation of an oxygen vacancy, the predicted CO vibration undergoes a redshift from 2134 cm^–1^ to 2085 cm^−1^ (Supplementary Table 1). When the reduced catalyst is treated under a dilute oxygen atmosphere, a partial oxidation is observed at around 323–373 K. However, before complete re-oxidation, CO is completely desorbed (Supplementary Figure 12) suggesting that there is a subsequent removal of CO from rhodium. Exposing the catalyst to a mixture of CO and O_2_ at 473 K leads to an absorption band at 2100 cm^−1^ (Fig. 4b), indicating a Rh^+^ species to be dominant in CO oxidation, in full agreement with operando XAS data, and thus provide further evidence to support the proposed catalyst cycle (Fig. 4d).

### Engineering more efficient CO oxidation catalysts

**Fig. 5:**
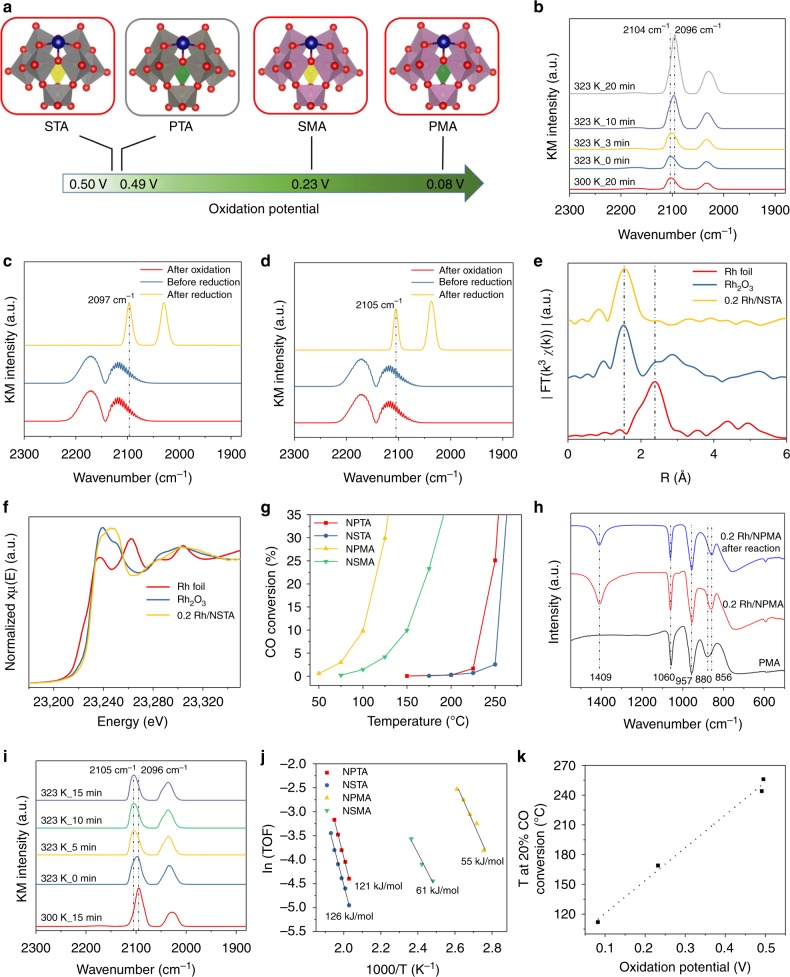
Ex and in situ characterization and catalytic performance of 0.2 Rh/NHPA. **a** Design principle for the development of HPA-based SACs based on the oxidation potentials of 4 HPAs (gray indicates the initially studied and red the new catalysts); CO DRIFT for **b** 0.2 Rh/NPMA, **c** 0.2 Rh/NSMA and **d** 0.2 Rh/NSTA, oxidation for (**c**, **d**) was carried out at 523 K for 1 h under 5% O_2_ and reduction was performed using 5% CO for 30 min at 373 K; **e** k^3^-weighted EXAFS spectra and **f** XANES for rhodium foil, Rh_2_O_3_ and 0.2 Rh/NSTA; **g** Temperature-activity curves for Rh catalysts (0.2 wt%, 100 mg), GHSV = 24,000 h^−1^ 1% CO/1% O_2_, balance Ar; **h** ATR-IR spectra for PMA, 0.2 Rh/NPMA before and after CO oxidation up to 300 °C (573 K); **i** in situ DRIFT spectra for the reoxidation of reduced 0.2 Rh/NPMA under 5% O_2_ atmosphere; **j** activation energies of the Rh catalysts (0.2 wt%) for CO oxidation; **k** correlation between T_20_ and oxidation potential of the support

From above, the reoxidation of the HPA support is the rate-limiting step during the CO oxidation. Designing a catalyst that has a lower barrier for the reoxidation should thus enhance the activity significantly. HPAs have been shown to be redox-tunable by changing the central atom of the whole Keggin unit (P or Si) or the central atom of the MO_4_ subunits (M = Mo, W) (Fig. 5a). To ensure full atomic dispersion of rhodium for all SACs based on those HPAs we synthesized them following the same procedure as reported previously with a weight loading of 0.2%. ATR-IR (Supplementary Figure 16), Raman spectroscopy (Supplementary Figure 17), and XRD (Supplementary Figure 18) confirms the intact structure of the heteropoly acids after adsorption of rhodium and precipitation as ammonium salt. We also confirmed the atomic dispersion of rhodium on the new catalysts by XAS and CO DRIFTS. CO DRIFTS analysis of the Rh/NPMA catalyst showed the occurrence of two peaks at 2104 and 2034 cm^−1^. Compared to rhodium on NPTA, the CO absorption wavelengths are slightly shifted towards lower wavenumbers, and rhodium supported on NPMA is reduced at slightly decreased temperatures compared to rhodium on NPTA (300 K vs. 323 K, Fig. 5b). For 0.2 Rh/NSTA and 0.2 Rh/NSMA, two peaks are observed with positions of the symmetric vibration peaks at 2105 for NSTA and 2097 cm^−1^ for NSMA, respectively, after a reductive pretreatment (Fig. 5c, d), suggesting the single-atom identity. Similar to the initially studied PTA-based catalyst, we observed a high white line intensity very close to the Rh_2_O_3_ sample and the absence of significant scattering contributions from shells above the first Rh–O contribution for 0.2 Rh/NSTA (Fig. 5e, f). The strong X-ray absorption by molybdenum close to the Rh X-ray edge prevented the XAS analysis for the two molybdenum-based samples. SEM analysis reveals that the morphology of the Rh_1_/NPMA catalyst is almost the same as for Rh_1_/NPTA with an EDX pattern revealing homogeneous distribution of Rh, Mo, P, N, and O whereas 0.2 Rh/NSTA and 0.2 Rh/NSMA exhibit a slightly different morphology (Supplementary Figures 19 and 20).

CO oxidation reactions were performed in a temperature range of 50–300 °C (323–573 K) with a GHSV of 24,000 h^−1^ and a partial pressure of each 0.01 bar for CO and O_2_. Distinct differences in the temperature behavior were observed, with the two Mo-based ones showing significant activity even at around 50 °C (323 K). The T_20_ values (temperature at which 20% CO conversion is achieved) for NPMA and NSMA are 112 °C (385 K) and 169 °C (442 K), respectively (Fig. 5g). In comparison, the tungsten-based catalysts only exhibit activity at higher temperatures of 256 °C (529 K) for silicotungstic acid (NSTA) and 244 °C (517 K) for NPTA (Fig. 5e). ATR-IR and Raman spectroscopy show that even after CO oxidation reaction at temperatures up to 300 °C (573 K), the heteropoly acid structure remains intact (Fig. 5h) and no formation of metal oxides was observed. In situ DRIFTS studies reveal that the reoxidation of reduced rhodium atoms on PMA occurs after being exposed to O_2_ at temperatures as low as 323 K for around 5 min indicated by a shift of the CO vibration from 2096 cm^−1^ back to 2105 cm^−1^ (Fig. 5i). The activation energies for 0.2 Rh/NPTA and 0.2 Rh/NSTA are very similar at 126 and 121 kJ/mol respectively whereas 0.2 Rh/NPMA exhibits a significantly lower activation energy of 55 kJ/mol in accordance with the significantly higher CO oxidation activity (Fig. 5j). A CO oxidation reaction test with 0.9 Rh/NPMA reveals an even lower T_20_ value (82 °C, 355 K) as compared to its lower weight loading counterpart (Supplementary Figure 21).

Although it proves difficult to experimentally determine the redox potential of solid catalysts, oxidation potentials of various HPAs have been determined based on electrochemical measurements in solution^61^. We found that the T_20_ values for CO oxidation indeed correlates very well with the oxidation potentials (Fig. 5k) proving the predictive power derived from the mechanistic understanding of CO oxidation SACs, i.e., the redox tunability of HPAs can be used synergistically to engineer enhanced CO oxidation catalysts.

## Discussion

Mechanistic elucidations are commonly employed to develop an improved understanding and to guide more intelligent catalyst design. In situ and operando spectroscopic studies represent some of the most promising approaches to investigate the active site and structural dynamics of heterogeneous catalysts under reaction conditions. Particle-based catalysts are well investigated but suffer from spectroscopic contributions from spectator species especially for surface-insensitive techniques such as XAS. SACs offer an enhanced platform as all metal species potentially contribute to the catalytic reaction, but metal oxides supported isolated atoms still suffer from a large heterogeneity. Supports that offer a much more limited number of binding sites with different adsorption energies benefits the homogeneity of metal species and the accuracy of spectroscopic investigations.

In the current work, we have demonstrated via an integrated operando/in situ XAS, XPS, and DRIFTS study that the atomically dispersed Rh-catalyzed CO oxidation follows a rather unconventional M–vK mechanism with the direct contribution of the heteropoly acid support. All the intermediates shown in Fig. 4d are confirmed by spectroscopy, where Rh undergoes a cycle between Rh^3+^ and Rh^+^ and the structure with oxygen vacancy is the dominant working state of the catalyst with the reoxidation of the support as the rate-controlling step. This study adds solid experimental evidence to the general understanding of how CO and O_2_ react over a single-atom site that is currently under debate. Stable, atomically dispersed metal sites on structurally well-defined sites particularly suitable for operando/in situ spectroscopy studies can be applied to reveal the reaction mechanisms for a range of different reactions, providing clues on how atomically dispersed catalysts work at a molecular level. As shown here, a combination of spectroscopic techniques, each of which provides information concerning an integral part of CO oxidation in a SAC system, provides a complete picture of a reaction mechanism. This enhanced mechanistic understanding, then, enables us to engineer supported Rh SACs that exhibit a significantly higher activity compared to the previously reported NPTA-supported Rh SAC.

## Methods

### Catalyst synthesis

The precipitated heteropoly acid ((NH_4_)_3_PW_12_O_40_) was synthesized following a coprecipitation method^44^. A solution of NH_4_NO_3_ (0.075 mol/L) and Rh(NO_3_)_3_ (appropriate amount for the desired weight loading), in 30 mL DI water was added at 1 mL/min to an aqueous solution of heteropoly acid (30 mL, 0.025 mol/L) under ice cooling and stirring (1200 rpm) after which a colloidal solution of precipitated Rh/NHPA formed. Under ice cooling and stirring (1200 rpm), the solution was aged for 5 h and then centrifuged (4 min, 12,200 × *g*) to obtain a colorless solid. After washing the solid three times with water, the product was dried. For STA and SMA, no precipitate formed after the addition of ammonium salt and thus, water was removed by freeze-drying and the catalyst was used without further washing steps.

### Catalyst characterization

We used an iCAP 6000 series inductively-coupled plasma optical emission spectrometry instrument to determine the weight loading of the catalysts after digestion in aqua regia overnight at 353 K. X-ray diffraction pattern were recorded on a Bruker D8 Advance X-ray diffractometer between 5 and 80°. Attenuated total reflection infrared spectroscopy was done using a Thermo Scientific Nicolet iS50 FT-IR spectrometer between 525 and 4000 cm^−1^ with a spectral resolution of 4 cm^−1^. Solid state Raman spectroscopy was performed at room temperature on a Horiba Jobin Yvon Modular Raman Spectrometer using a 532 nm excitation laser, a ×100 objective, 1% filter and 1200 mm^−1^ grating. A silicon wafer was used to calibrate the Raman shift. Transmission electron microscopy and scanning electron microscopy images were recorded on a JEM 2100F (JEOL) and on a JEM-6700F (JEOL) microscope, respectively.

### Operando and ex situ XAS experiments

Catalyst pellets were made from a thoroughly ground mixture of 300 mg catalyst with 70 mg boron nitride. Sample measurement details are the same as provided in a previous paper^62^. Wavelet analysis was performed using the Morlet wavelet transform procedure with values for κ and σ of 5 and 1, respectively^63^. After collecting the spectra at room temperature under ambient conditions, an atmosphere of dilute CO (0.05 bar partial pressure in He) was applied in a high-temperature operando cell specifically designed for measurements in the fluorescence mode. Dilute O_2_ was introduced at a partial pressure of 0.07 bar in He. After each measurement (duration of around 1 h), the catalyst was heated up rapidly within around 10 min and we waited for the temperature to reach a steady state (around 10 min) upon which another measurement was started. During the catalyst treatment with the mixed gas atmosphere (CO + O_2_, 0.025 bar partial pressure each in He), the reaction was performed in an excess of reactant gases to ensure that the reaction is conducted under purely kinetic limitations (Supplementary Figure 1). A similar procedure was used for the tungsten XAS measurements but another operando cell designed for measurements in the transmission mode was employed and the time for each measurement reduced to 15–20 min.

Ex situ Rh K-edge XAS measurements were carried out at a public beamline, BL14B2, SPring-8 (Japan Synchrotron Radiation Research Institute, Hyogo, Japan).

### DRIFTS experiments

In situ diffuse reflectance infrared Fourier transform (DRIFT) spectra were recorded using an iS50 FT-IR spectrometer with a mercury-cadmium-telluride (MCT) detector and a Praying Mantis^TM^ high-temperature reaction chamber with zinc selenide windows. For all spectra, 32 scans with a resolution of 4 cm^−1^ were recorded in a range of 1700–2350 cm^−1^. Powder samples were placed in a reaction cell (Harricks HV-DR2) and background spectra were taken under a flow of inert gas at different temperatures. All gases and gas mixtures were introduced at 40 mL/min and the partial pressures were 0.025 bar for CO and O_2_ for the individual gases and 0.025 bar each for the mixture of CO and O_2_ (balance Ar). For the oxygen treatment of the catalyst, the catalyst was subjected to a reductive treatment under dilute CO for 20 min at room temperature prior to the introduction of dilute O_2_.

Ex situ measurements were performed using a similar procedure as described above but after collecting background spectra under nitrogen atmosphere, dilute CO was introduced until a steady state was reached upon which nitrogen gas was introduced again to remove gas phase CO. For the oxidative treatment of the 0.2 Rh/NSTA and 0.2 Rh/NSMA catalysts, the catalyst powder was treated under 5% oxygen at 523 K for 1 h and cooled down to room temperature after which the CO treatment was done as mentioned above. Reductive treatment was done using 5% CO at 323 K for 30 min after which the catalyst was cooled back to room temperature and gas phase CO was removed by flushing nitrogen.

### In situ XPS experiments

Catalyst pellets were produced by thoroughly mixing and pressing 200 mg catalyst, 50 mg boron nitride, and 2 mg graphene nanoplatelets (750 m^2^/g) as internal standard. All binding energies were adjusted based on the most intense C 1s peak at 284.5 eV. The in situ XPS experiments were performed in the laboratory-based SPECS Phoibos 150 NAP-XPS (near ambient pressure XPS) package equipped with a twin anode X-ray source (SPECS XR50, Al Kα, hν = 1486.6 eV; Mg Kα, hν = 1253.6 eV) with a base pressure at 5 × 10^–10^ mbar. Two independent gas lines were connected to the NAP cell to let gas fill the cell to several mbar from ultra-high vacuum effectively in 2 min. The catalyst was loaded near the 300 µm nozzle of the NAP cell and illuminated to the X-ray through a 100 nm SiN window. The temperature-programmed mode was carried out in the NAP-XPS measurements: 0.5 mbar CO gas was introduced to the cell at room temperature and then the cell was heated from 300 K to 473 K in 5 steps. After that, the system was cooled down to room temperature using liquid nitrogen cooled nitrogen gas and the CO gas was pumped out to UHV. 0.5 mbar O_2_ gas was introduced to the system as following in the same procedures and the catalyst was exposed to the mixture of CO and O_2_ (with 0.5 mbar partial pressure each) gases at different temperatures. All the measurements were taken until the pressure and temperature were stable to reach the equilibrium conditions of the surface. The core-level spectra of W 4*f*_7/2_ were measured by using the Al Kα source with kinetic energies at around 1450 eV. The pass energy was set at 30 eV for each measurement.

### DFT calculations

Calculations were carried out with the local meta-GGA correlation functional M06L^64^ using Gaussian09 D.01 as has been employed for polyoxometalates^65^ earlier. All geometry optimizations and frequency calculations were done using the basis sets 6–31G**^66^ for the lighter elements (C, O, P) and the pseudopotential basis sets LANL2DZ^67^ for tungsten and rhodium. Each obtained optimized structure was checked to have the right number of imaginary frequencies. Images of the geometry-optimized structures were created using VESTA. CO vibrations were predicted using the same functional and basis set with a scaling factor of 0.972^68^.

### Simulations of XANES spectra

The Rh K-edge XANES simulations were conducted using the ab initio FEFF 8 program^69^. The structural models from DFT calculations were used for the atomic coordinates in the FEFF input cards. A slight modification (0–2%) of the bond distances using the RMULTIPLIER card was allowed to optimize the XANES simulations. The simulated XANES spectra were plotted using the normalized E0 scale (E-E0). The E0 value of the simulated XANES was set at 0 eV by calibrating the shift of calculated Fermi levels from the FEFF SCF calculations.

### CO oxidation reaction

CO oxidation reactions were performed in a stainless steel tubular plug flow reactor. A volume of 100 mg catalyst was charged into the reactor surrounded by quartz wool and the reactor was heated inside a tube furnace with an external thermocouple. Catalysts were pretreated under 5% O_2_ in Ar for 30 min at 250 °C (523 K) with a heating rate of 5 K/min. A mixture of 2.5% CO and 2.5% O_2_ balanced with Ar was introduced and the tube was heated to the desired starting temperature. Steady-state conversion and yield measurements were performed after reaching steady-state after 30 min reaction using an Agilent 7890B gas chromatograph with a TCD detector. For activation energy measurements, the CO conversion was kept below 25%.

## Supplementary information

Supplementary Information

Peer Review

## Data Availability

The data that support the findings of this study are available from the corresponding authors upon reasonable request.
