# Supplementary material for: In situ spectroscopy-guided engineering of rhodium single-atom catalysts for CO oxidation
Source: Nat Commun. 2019 Mar 22;10:1330. doi: 10.1038/s41467-019-09188-9 (PMC6430772; doi:10.1038/s41467-019-09188-9)
Supplement: Supplementary file 1 — Supplementary Information [file 41467_2019_9188_MOESM1_ESM.pdf]

Supporting Information

In situ Spectroscopy-Guided Engineering of Rhodium Single-Atom Catalysts for CO Oxidation

Hülsey et al.

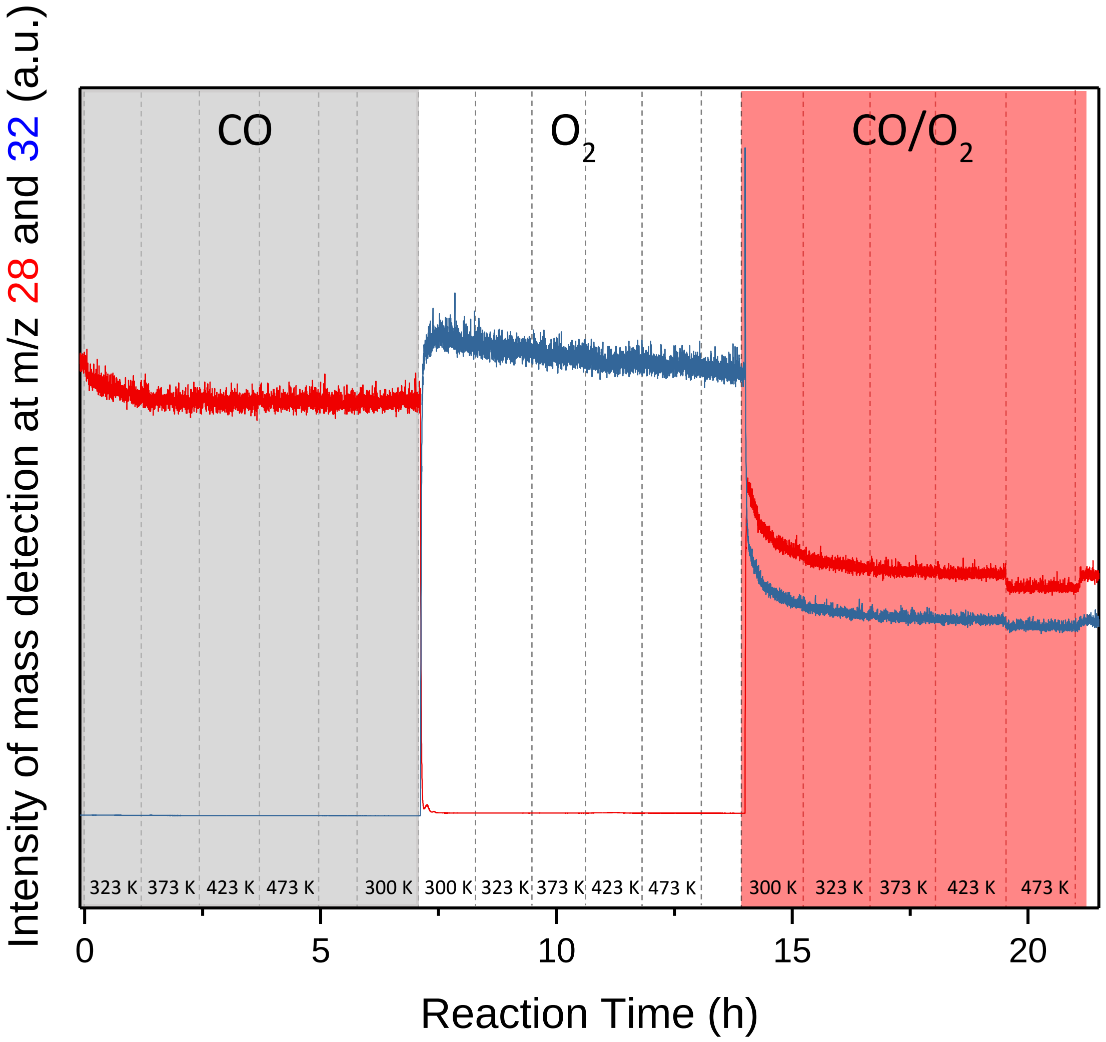

Supplementary Figure 1 | *Operando* detection of CO and O_2. Mass spectrometry results during the *operando* XAS experiment at the Rh K-edge for m/z 28 (CO, red) and 32 (O_2, blue). The temperature steps are indicated by vertical lines. The respective gas treatments are indicated by different colors.

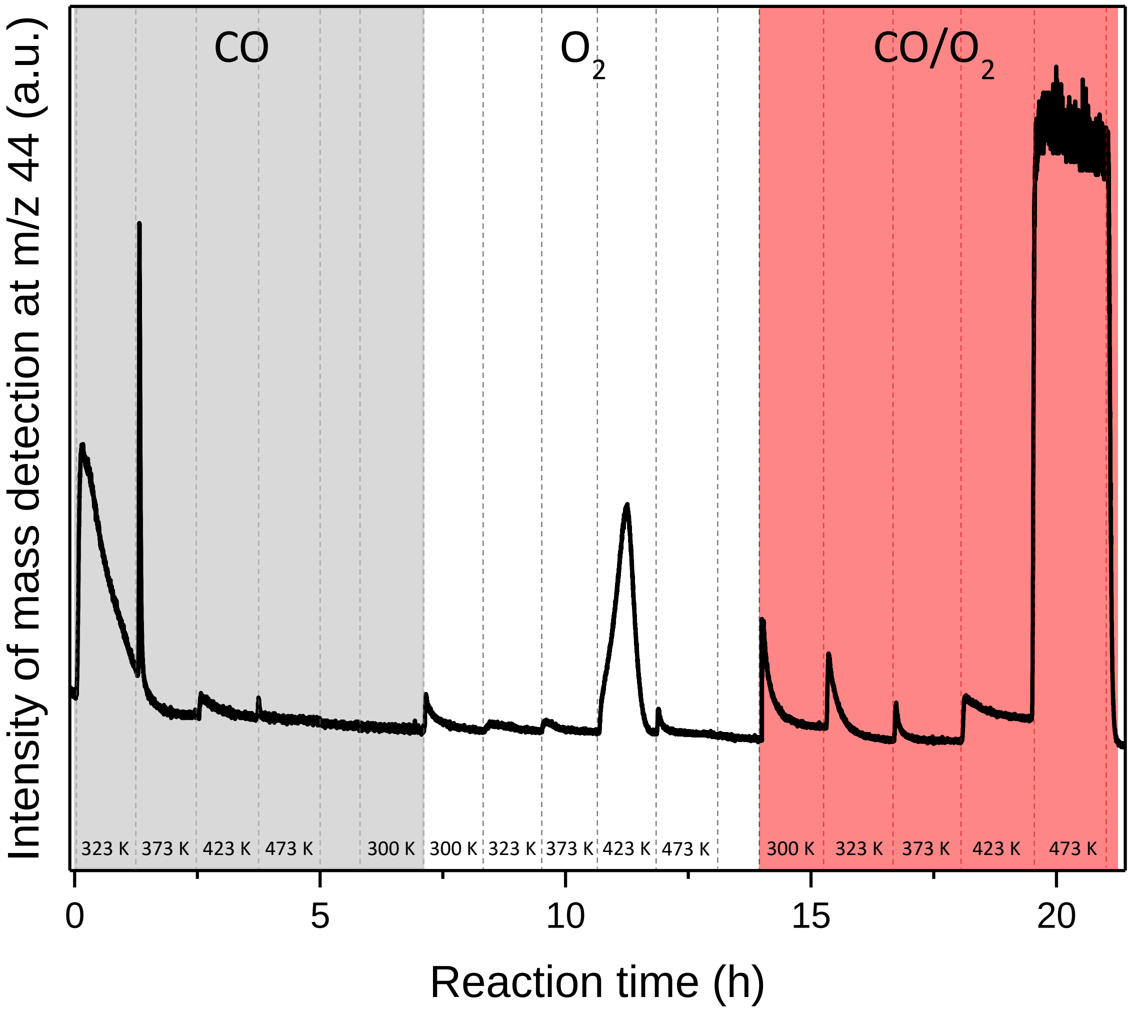

Supplementary Figure 2 | Operando detection of CO_2. Mass spectrometry result during the operando XAS experiment at the Rh K-edge for m/z 44 (CO_2). The temperature steps are indicated by vertical lines. The respective gas treatments are indicated by different colors and follow the same order as indicated in **Fig. S 1**. The increase in CO_2 detection in the beginning of the CO/O_2 treatment can be ascribed to an increase in the background (omitted here for clarity).

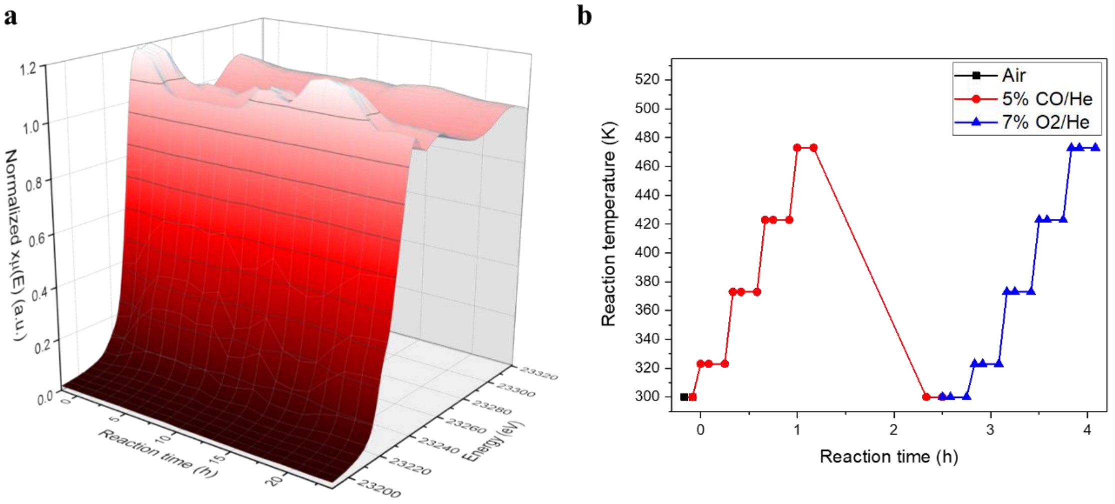

Supplementary Figure 3 | Course of the *operando* XAS experiments. (a) Dynamics of the XAS spectra during the operando experiment at the Rh K-edge throughout the experiment; (b) Course of the XAS experiment at the W L_{III}-edge in transmission mode. The catalyst was kept at the respective temperature for 5 minutes to ensure a constant temperature. Each XAS experiment in transmission mode (W L_{III}-edge) took about 10 minutes.

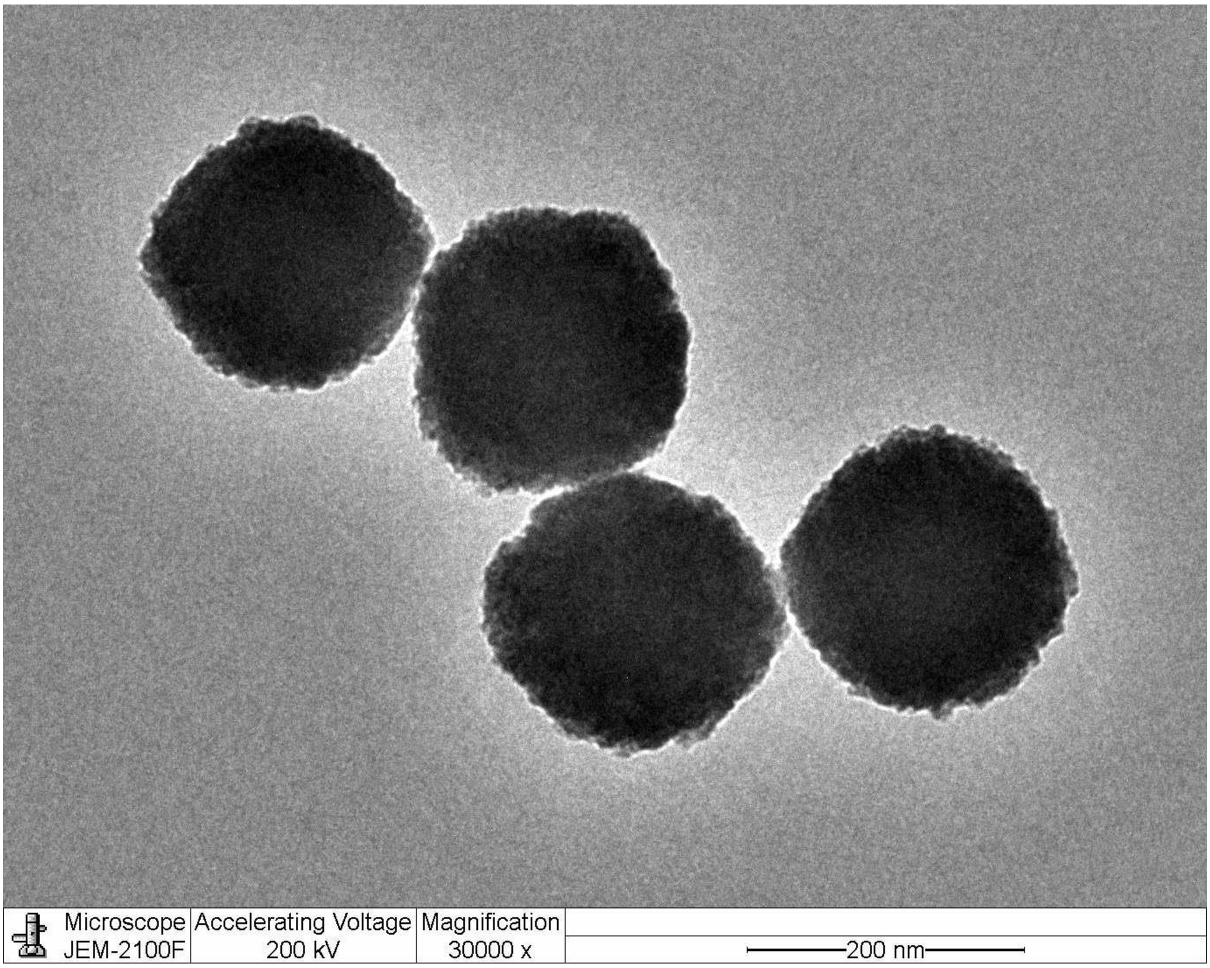

Supplementary Figure 4 | Microscopy image. TEM image of the Rh_1/NPTA catalyst as synthesized.

a

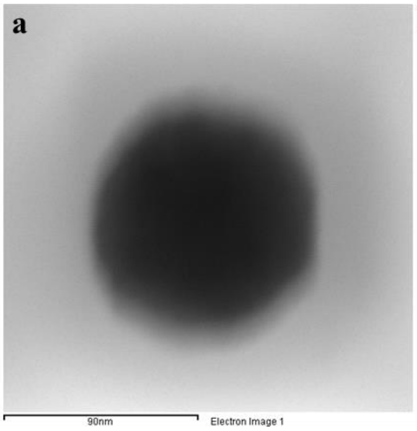

b

Spectrum processing:
Peaks possibly omitted: 3.700, 6.401 keV

Quantitation method: Cliff Lorimer thin ratio section.
Processing option: All elements analyzed (Normalised)
Number of iterations = 1

Standardless

Element	Weight%	Atomic%
O K	16.31	67.97
P K	1.18	2.48
Rh L	0.83	0.52
W L	81.69	29.03
Totals	100.00	

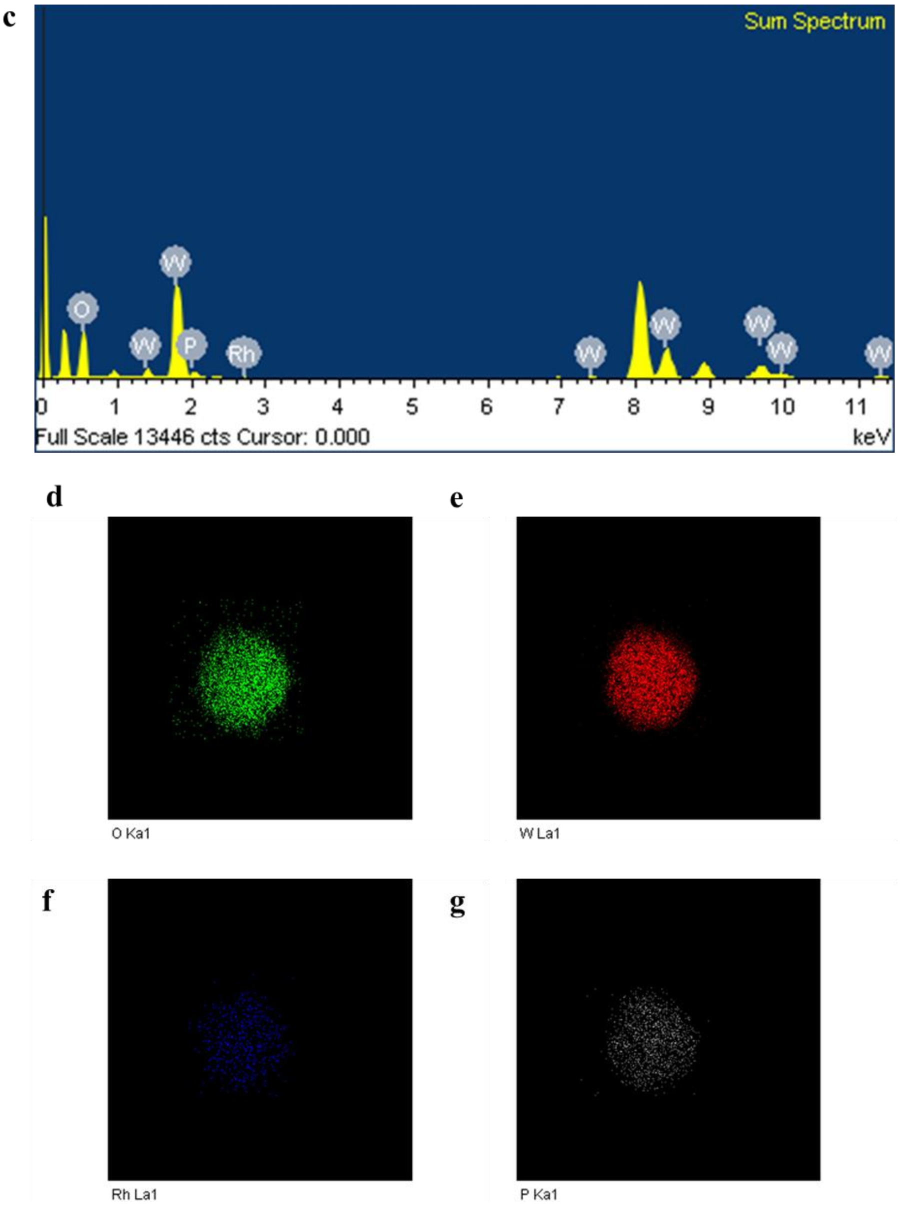

Supplementary Figure 5 | EDX analysis of Rh$_1$/NPTA. (a) TEM image of the catalyst particles used for EDX analysis. (b) Spectrum collection parameters and atomic percentages. (c) Sum spectrum of the catalyst particle as depicted in (a). EDX mapping for (d) oxygen, (e) tungsten, (f) rhodium and (g) phosphorus

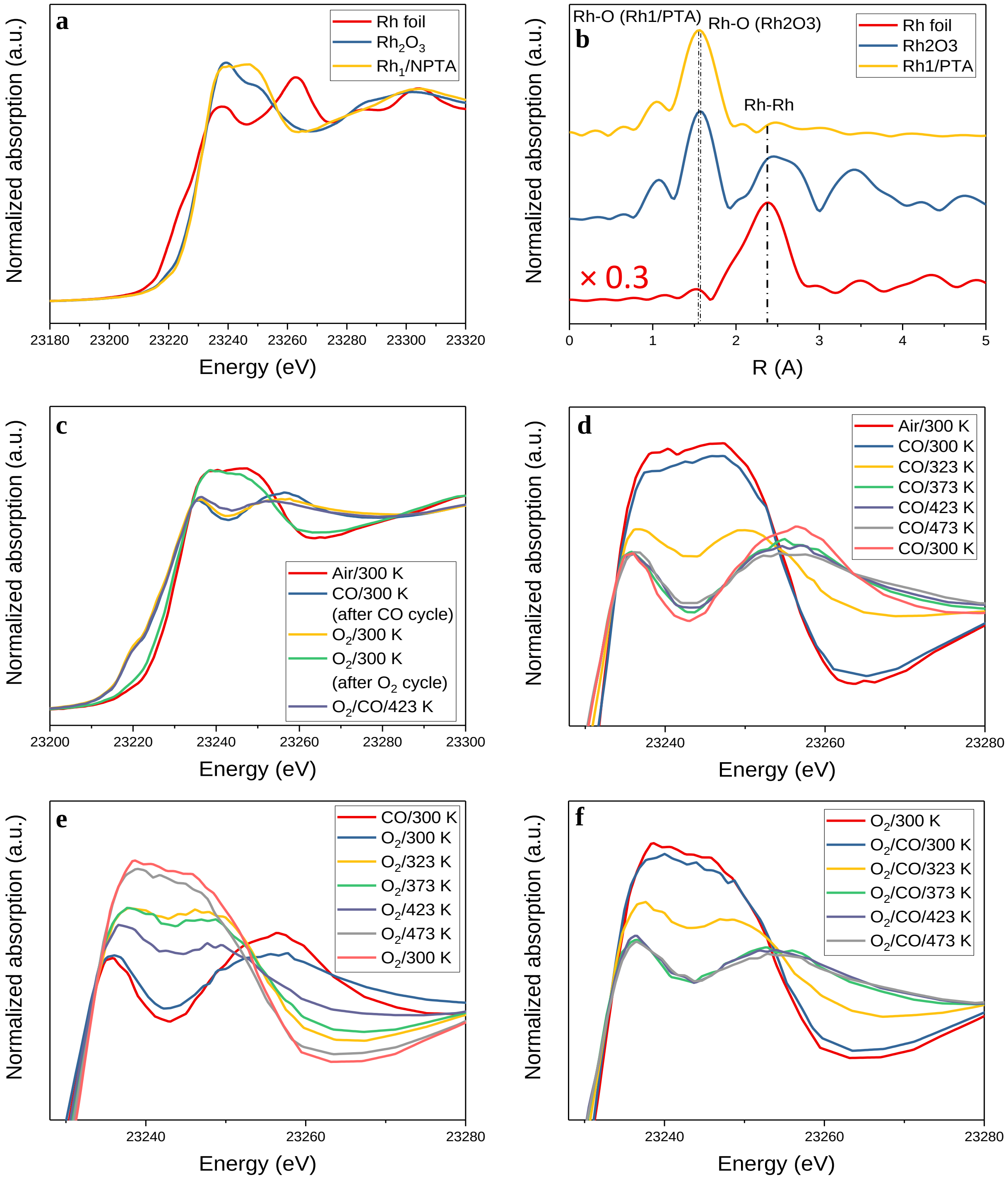

Supplementary Figure 6 | Overview over Rh K-edge XAS results. (a) Normalized X-ray absorption spectra and (b) k^3-weighted EXAFS spectra on the Rh K- edge for rhodium foil, Rh_2O_3 and Rh_1/NPTA; (c), (d), (e) and (f) XANES spectra of the catalyst under different temperatures and gas atmospheres.

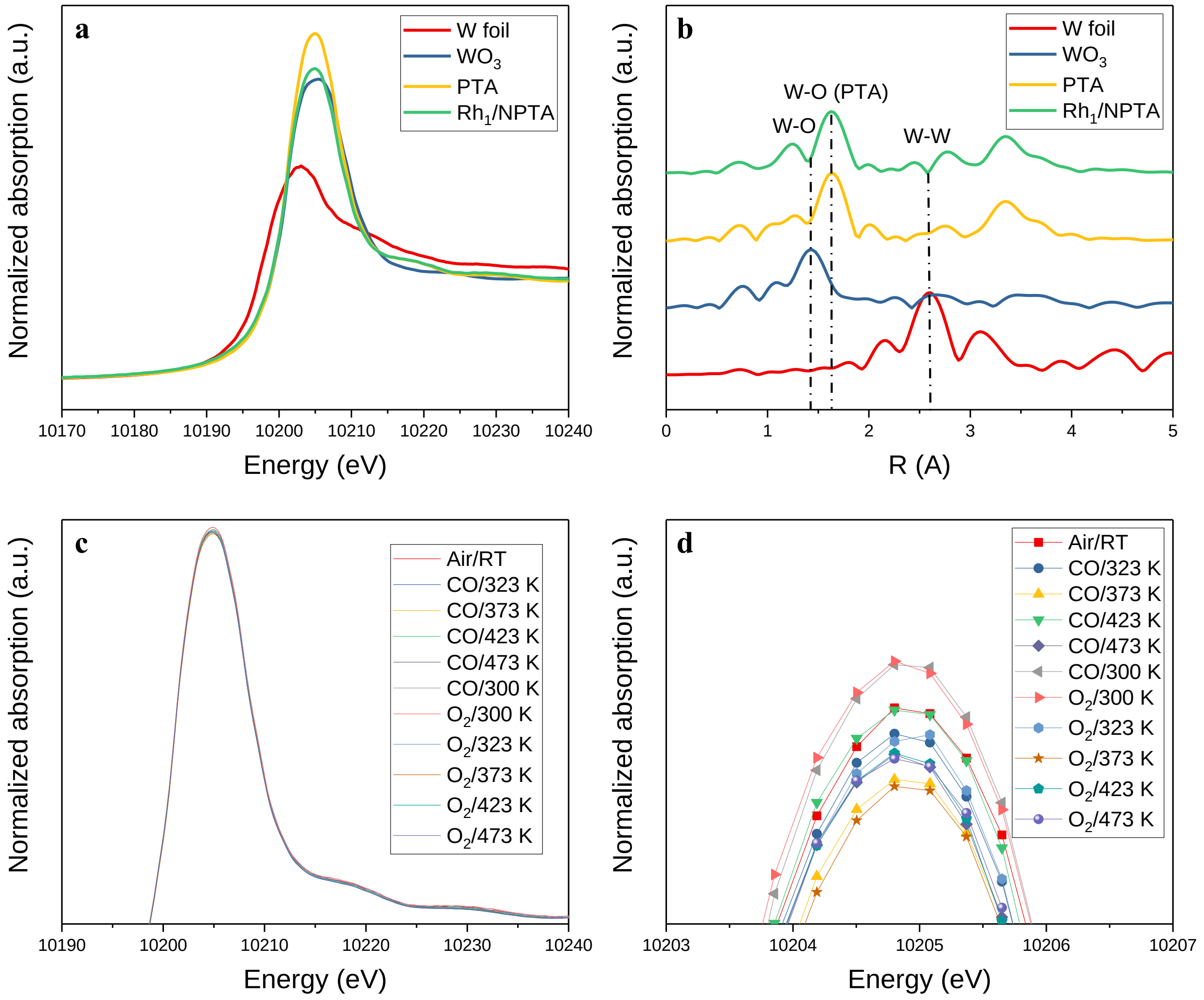

Supplementary Figure 7 | Overview over W L$_{III}$-edge XAS results. (a) Normalized X-ray absorption spectra and (b) k^3-weighted EXAFS spectra on the W L$_{III}$-edge for tungsten foil, WO_3, phosphotungstic acid (PTA) and Rh_1/NPTA; (c) XANES and (d) detailed XANES spectrum (see red frame in (C)) at the W L$_{III}$-edge for the catalyst under different temperatures and gas atmospheres.

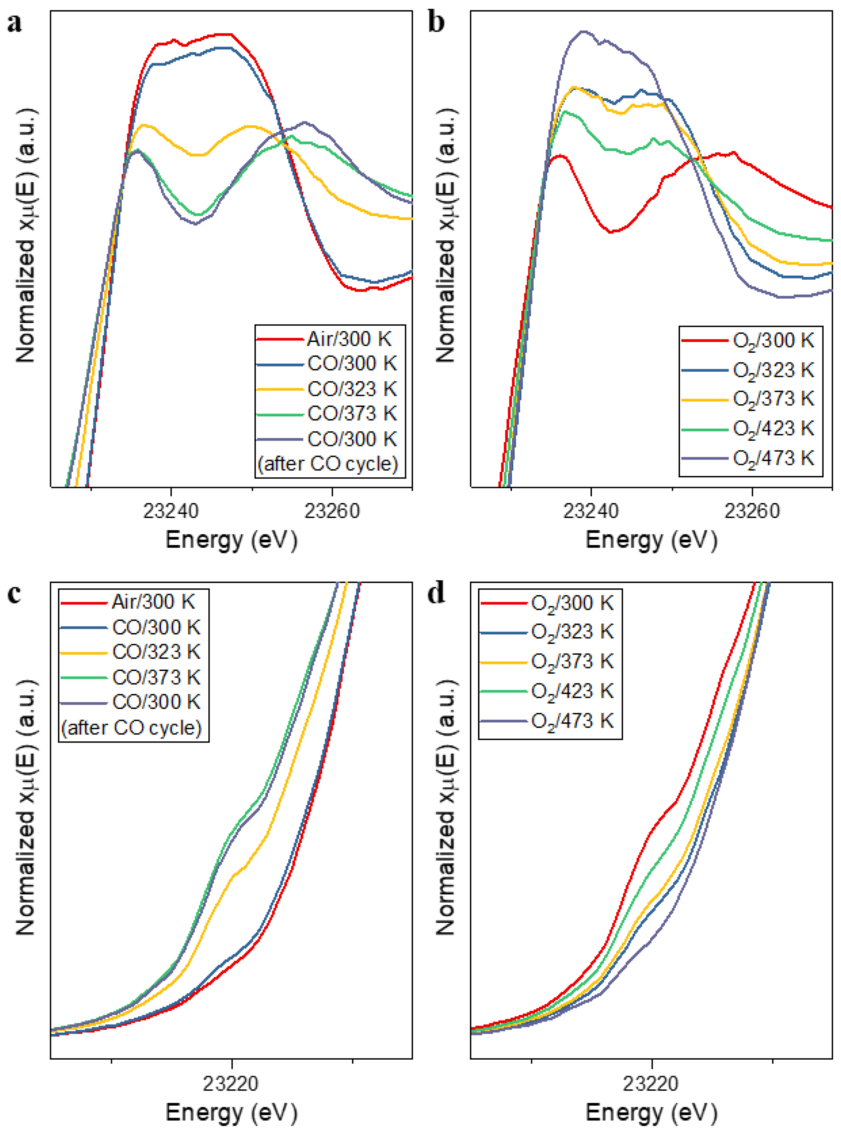

Supplementary Figure 8 | *Operando* Rh K-edge XANES spectra. Normalized X-ray absorption spectra at the Rh K-edge at different temperatures under CO (a) or O_2 (b) atmosphere with (c & d) a special emphasis on the pre-edge region.

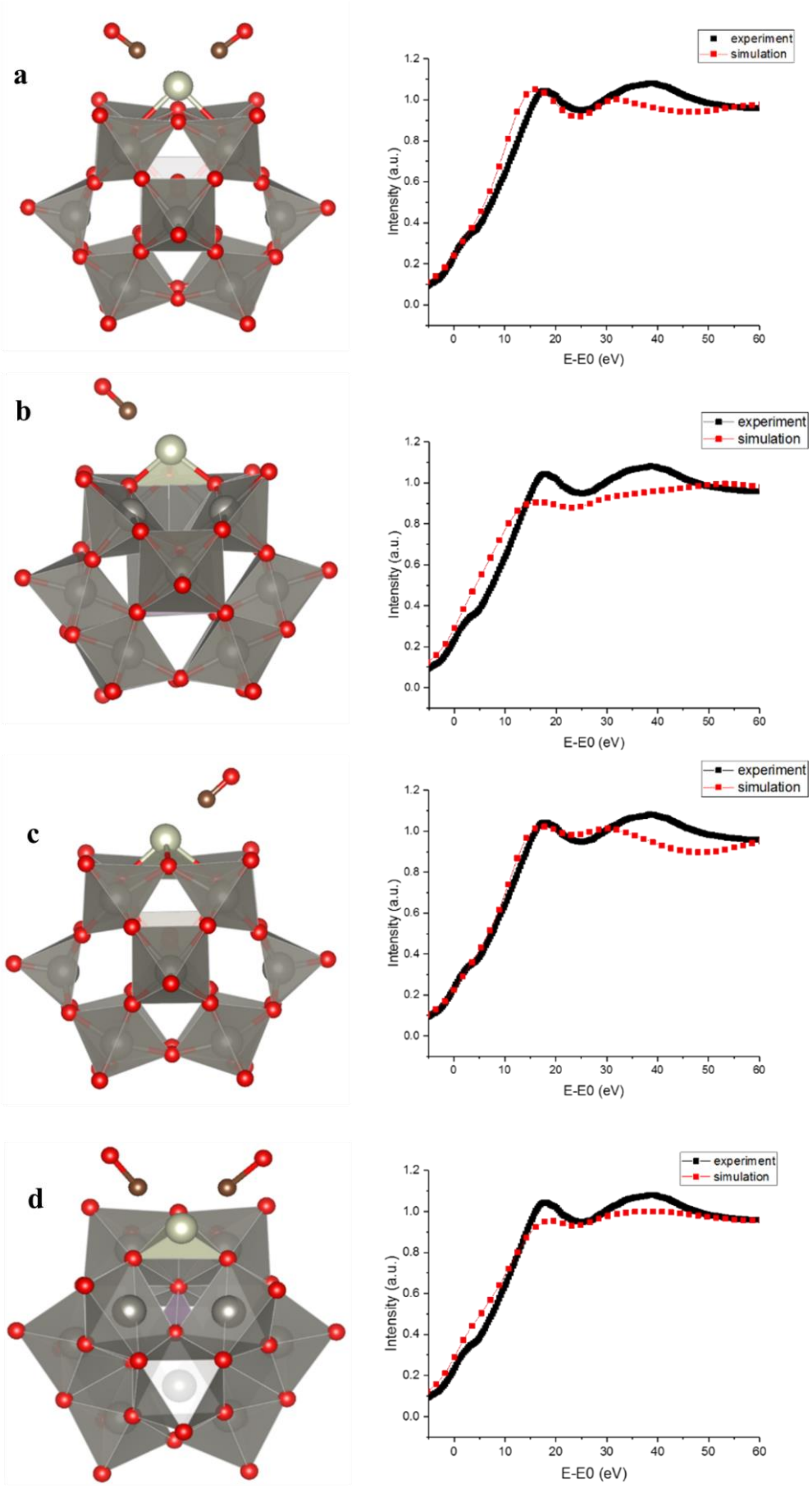

Supplementary Figure 9 | DFT models and simulated XANES spectra. Models for the DFT-optimized structures, original and simulated XANES spectrum for the (a) structure with 4 coordinating oxygen atoms and two CO molecules; (b) structure with 3 coordinating oxygen atoms (oxygen vacancy) and one CO molecule; (c) structure with 4 coordinating oxygen atoms and two CO molecules; (d) structure with three coordinating oxygen atoms (oxygen vacancy) and two CO molecules.

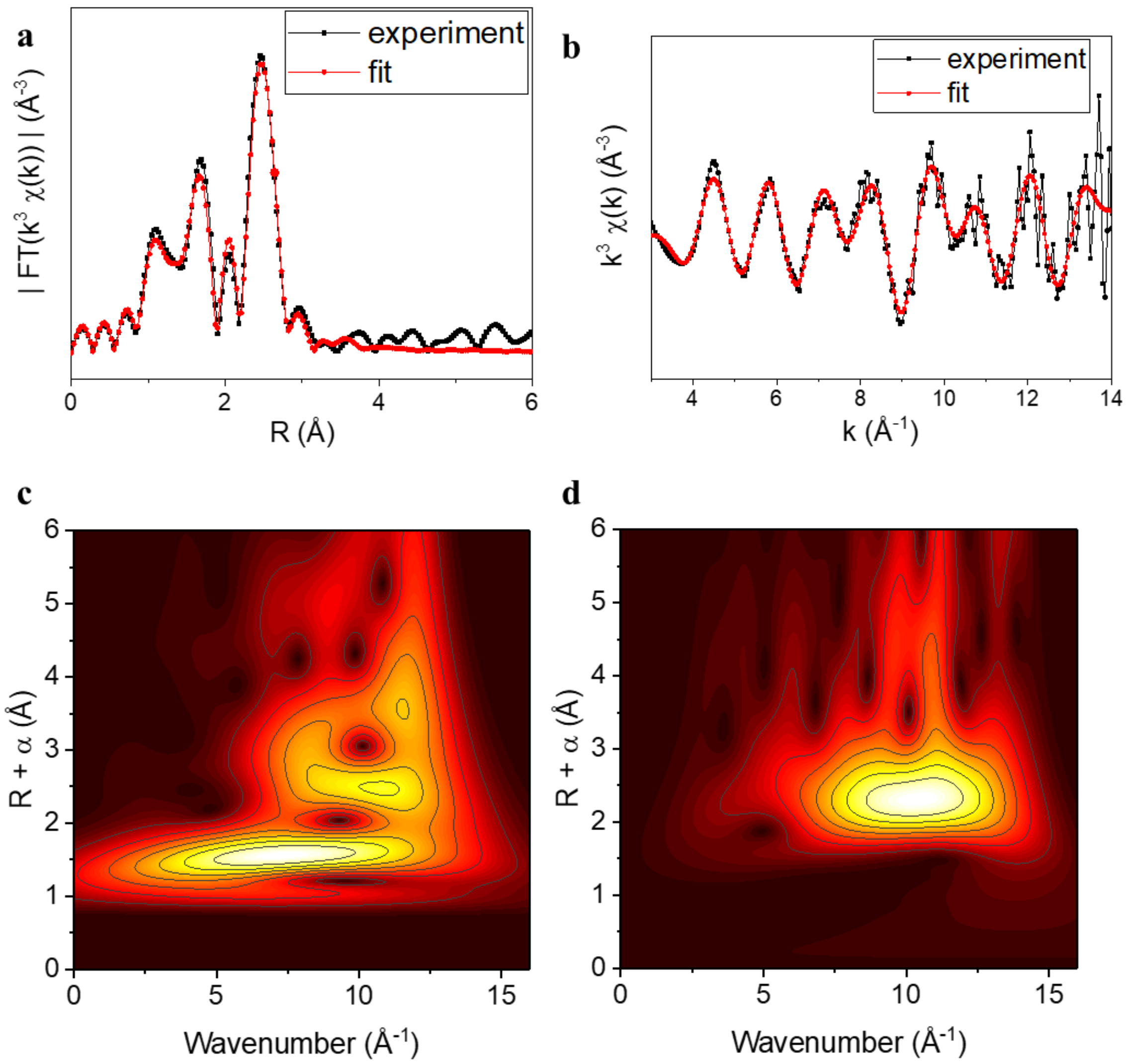

Supplementary Figure 10 | Rh K-edge EXAFS fitting and wavelet transformation. Fitting of the EXAFS spectrum of the catalyst after reduction with CO during the *in situ* experiment in the (a) R and (b) k space; Morlet wavelet transform for a (c) Rh_2O_3 and (d) rhodium foil reference material.

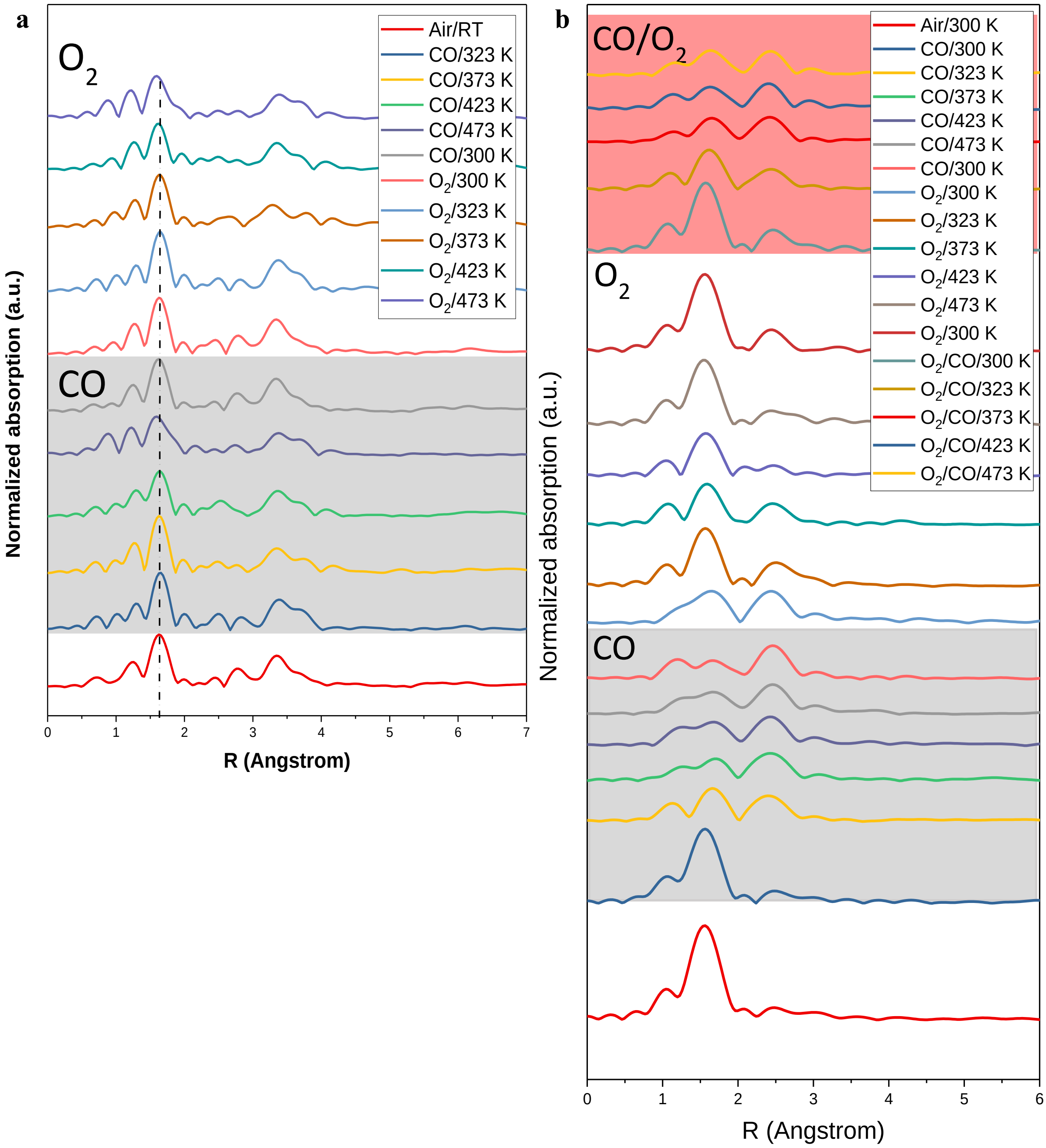

Supplementary Figure 11 | *Operando* Rh K-edge and W LIII-edge EXAFS spectra. (A) k^3-weighted EXAFS spectra of the catalyst at different temperatures and gas atmospheres at the W-LIII edge; (B) k^3-weighted EXAFS spectra of the catalyst at different temperatures and gas atmospheres at the Rh-K edge.

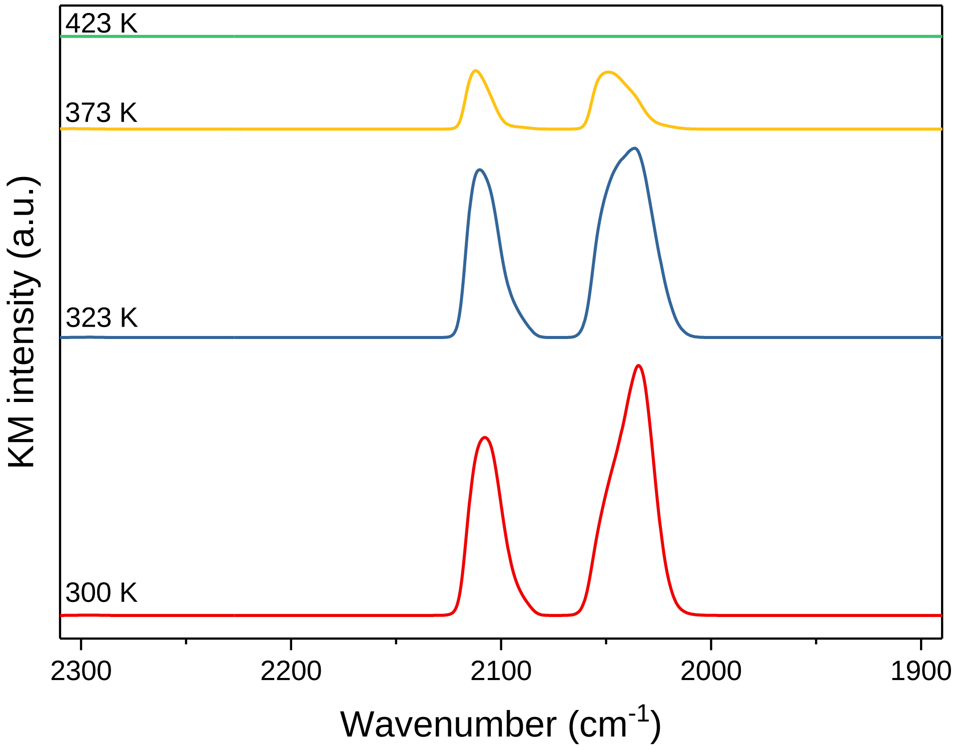

Supplementary Figure 12 | *In situ* DRIFT spectroscopy. *In situ* DRIFT spectra of the pre-reduced catalyst under 5% O_2 atmosphere at different temperatures.

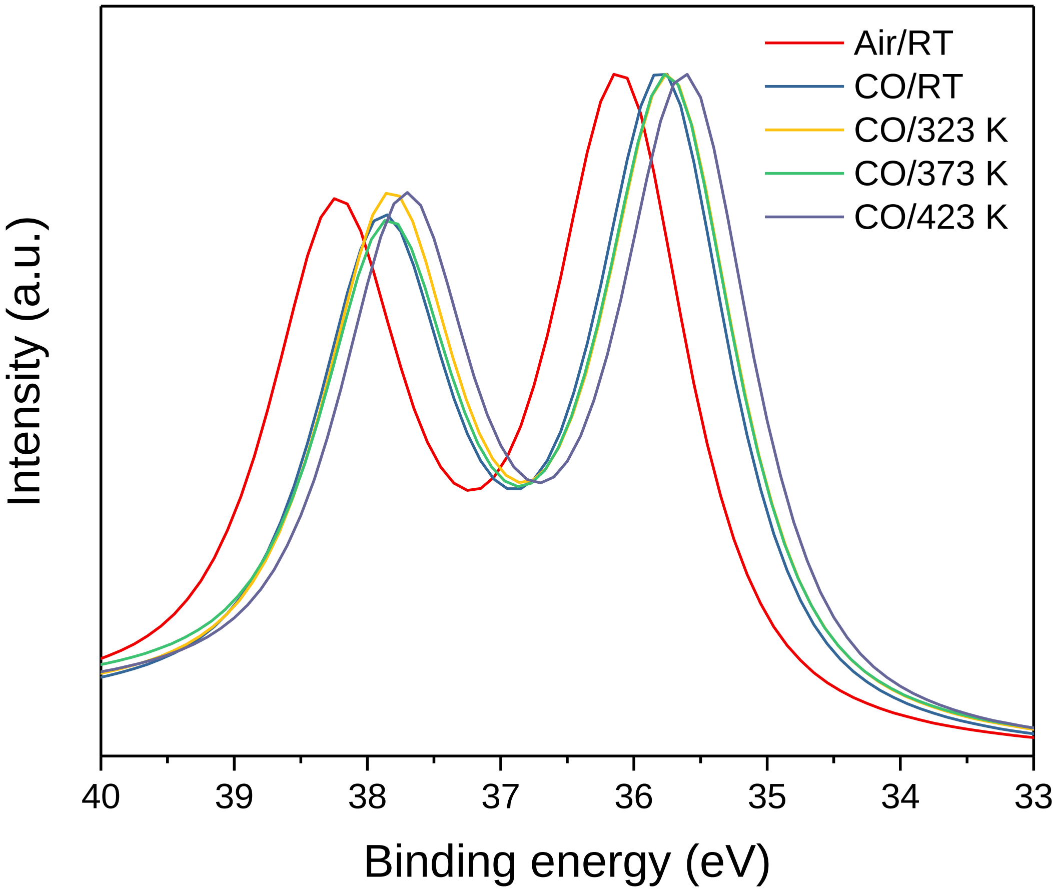

Supplementary Figure 13 | *In situ* XPS spectroscopy under air and CO. *In situ* XPS spectra of the catalyst under CO atmosphere at different temperatures.

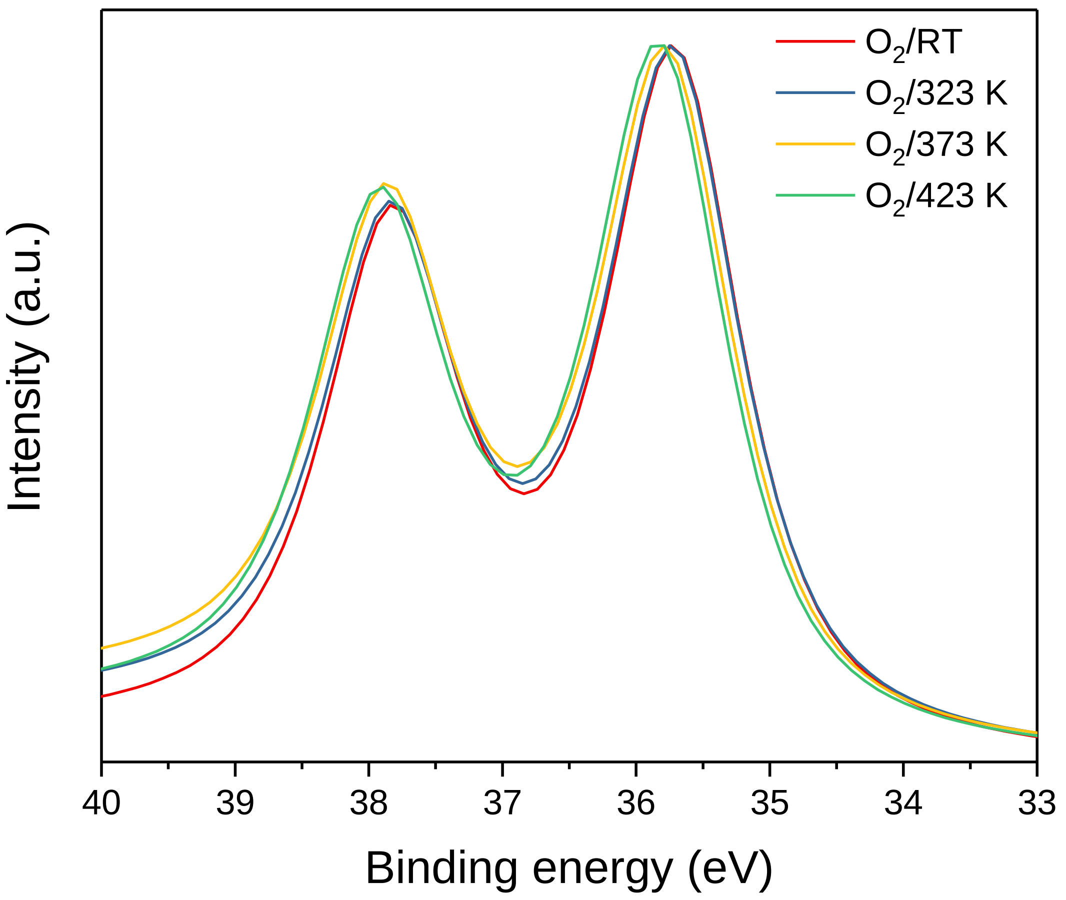

Supplementary Figure 14 | *In situ* XPS spectroscopy under O_2. *In situ* XPS spectra of the reduced catalyst under O_2 atmosphere at different temperatures.

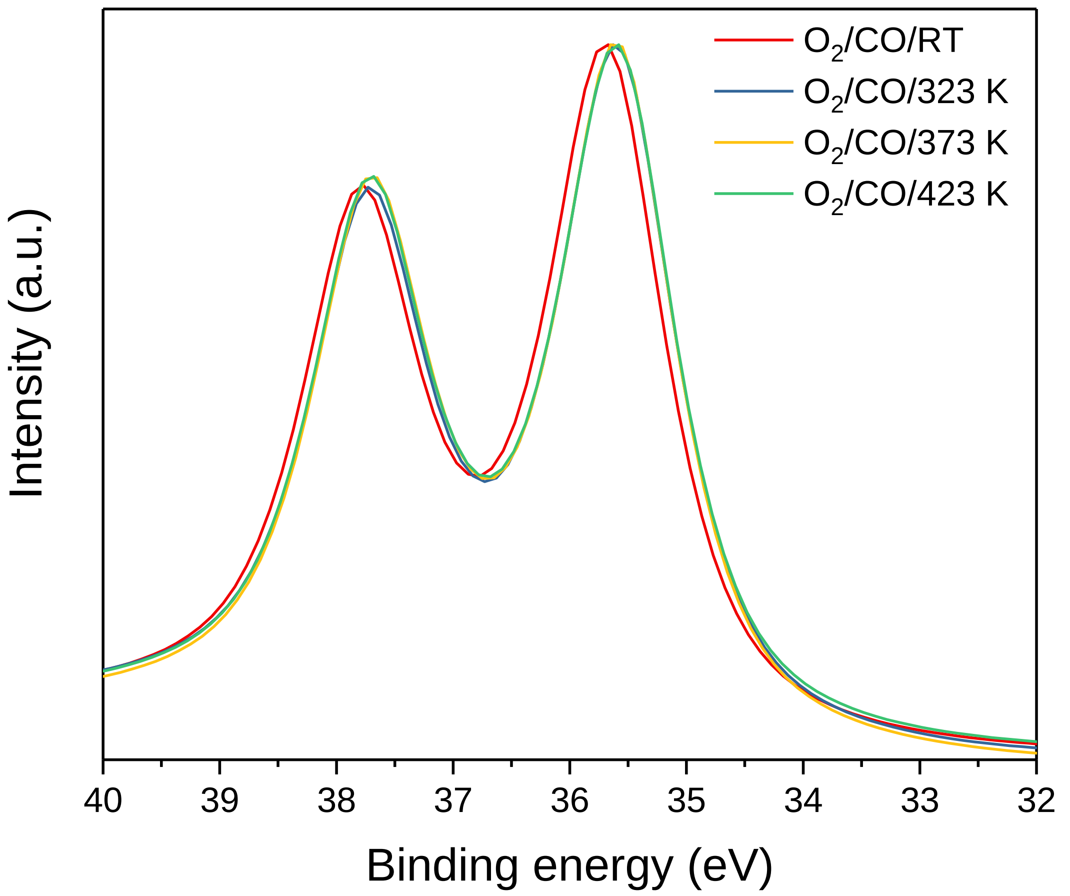

Supplementary Figure 15 | *In situ* XPS spectroscopy under CO/O_2. *In situ* XPS spectra of the reduced catalyst under O_2 atmosphere at different temperatures.

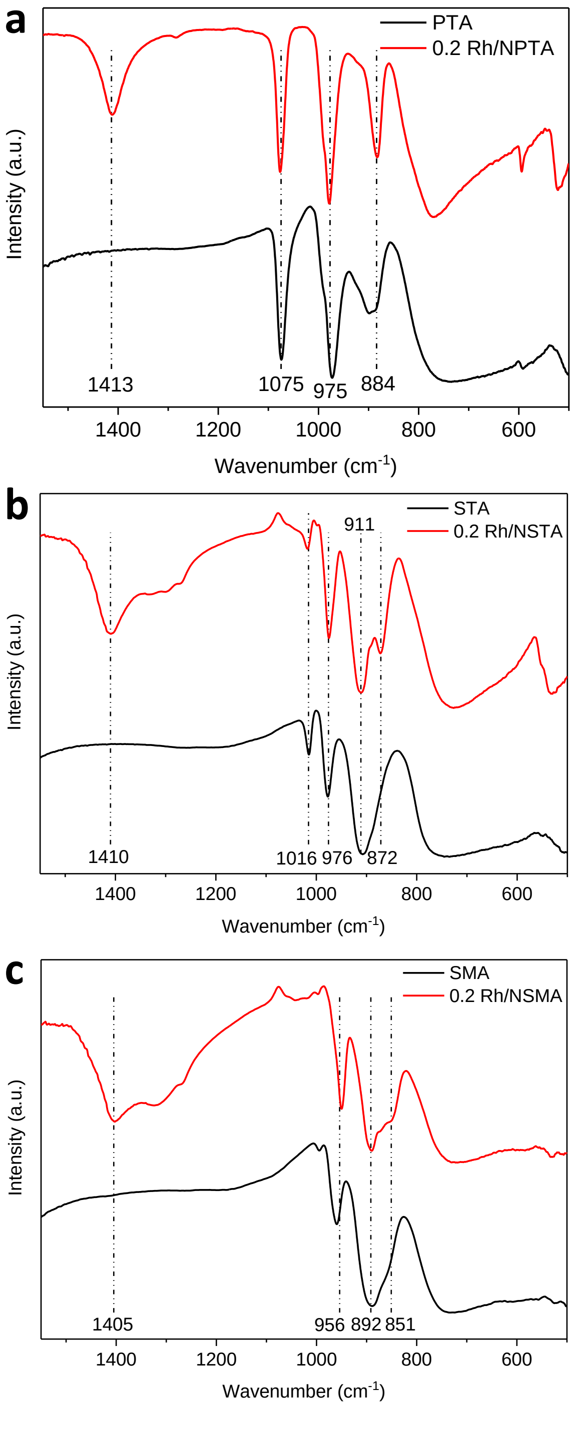

Supplementary Figure 16 | ATR-IR of different catalysts. ATR-IR spectra of **a** PTA and 0.2 Rh/NPTA, **b** STA and 0.2 Rh/NSTA and **c** SMA and 0.2 Rh/NSMA.

Only ATR-IR spectroscopy shows a shifted peak position for PTA and PMA (phosphomolybdic acid) from 891 and 885 to 884 and 856 cm^{-1} respectively and the enhancement of a peak shoulder at 872 cm^{-1} for STA (silicotungstic acid) and 851 cm^{-1} for SMA (silicomolybdic acid). The appearance of additional peaks for heteropoly acids has been predicted previously using DFT-based vibrational analysis of single atoms on the heteropoly acid Keggin structure.[1] The occurrence of a peak located at 1410 cm^{-1} can be ascribed to N-H vibrations from ammonium ions in the HPA salts.

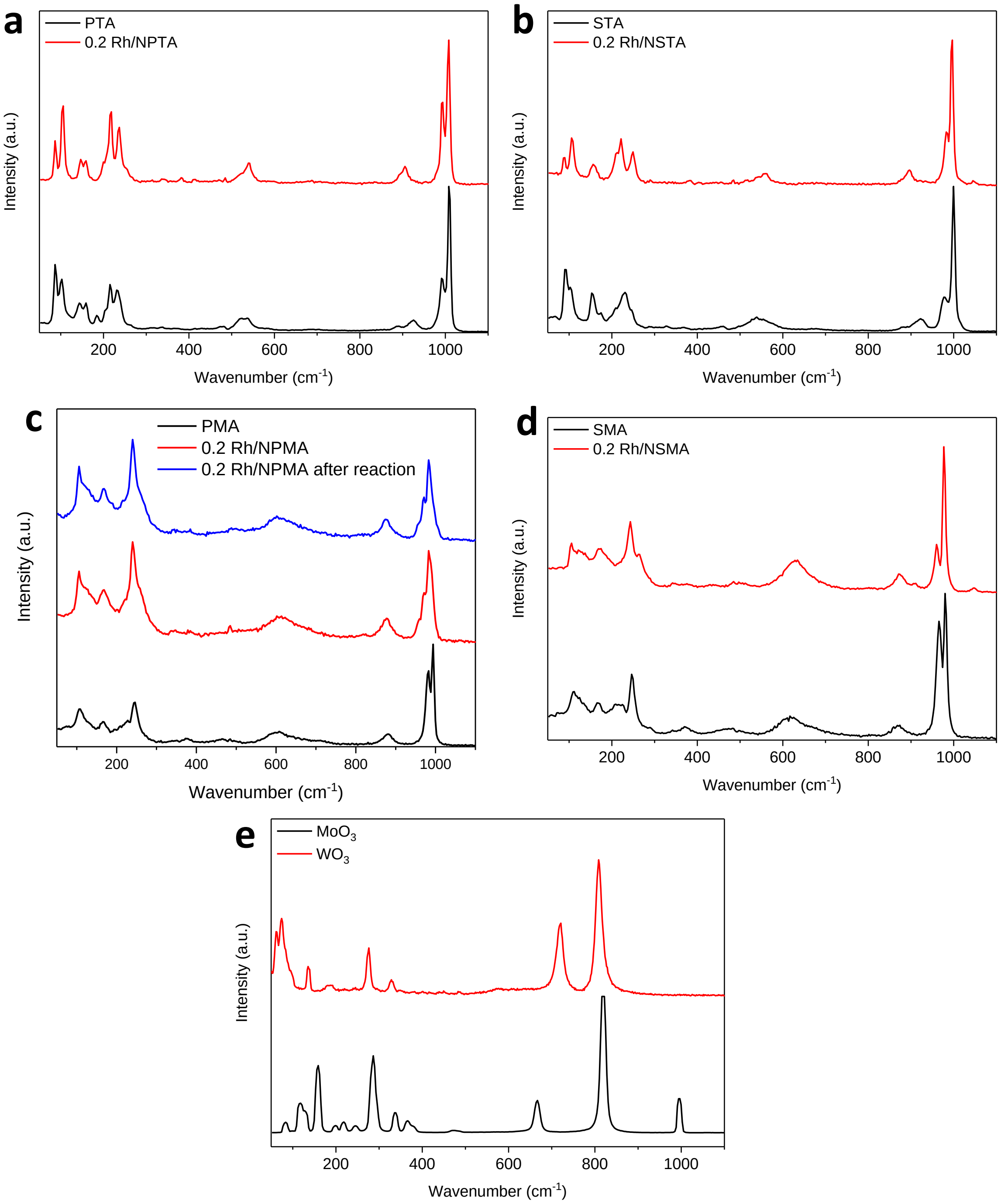

Supplementary Figure 17 | Raman spectra of different catalysts. Raman spectra of **a** PTA and 0.2 Rh/NPTA, **b** STA and 0.2 Rh/NSTA, **c** PMA and 0.2 Rh/NPMA before and after CO oxidation reaction at up to 300 °C, **d** SMA and 0.2 Rh/NSMA and **e** MoO_3 and WO_3 at 532 nm excitation wavelength.

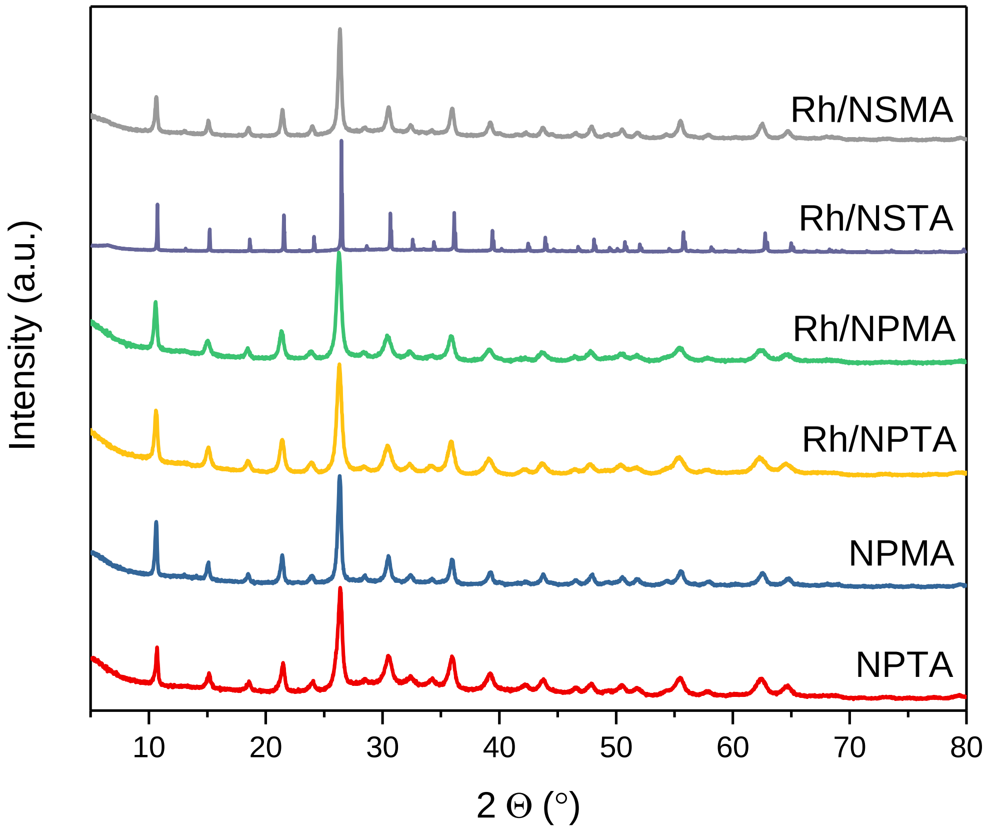

Supplementary Figure 18 | XRD pattern. (P)XRD pattern of different 0.2 Rh/NHPA catalysts, NPMA and NPTA.

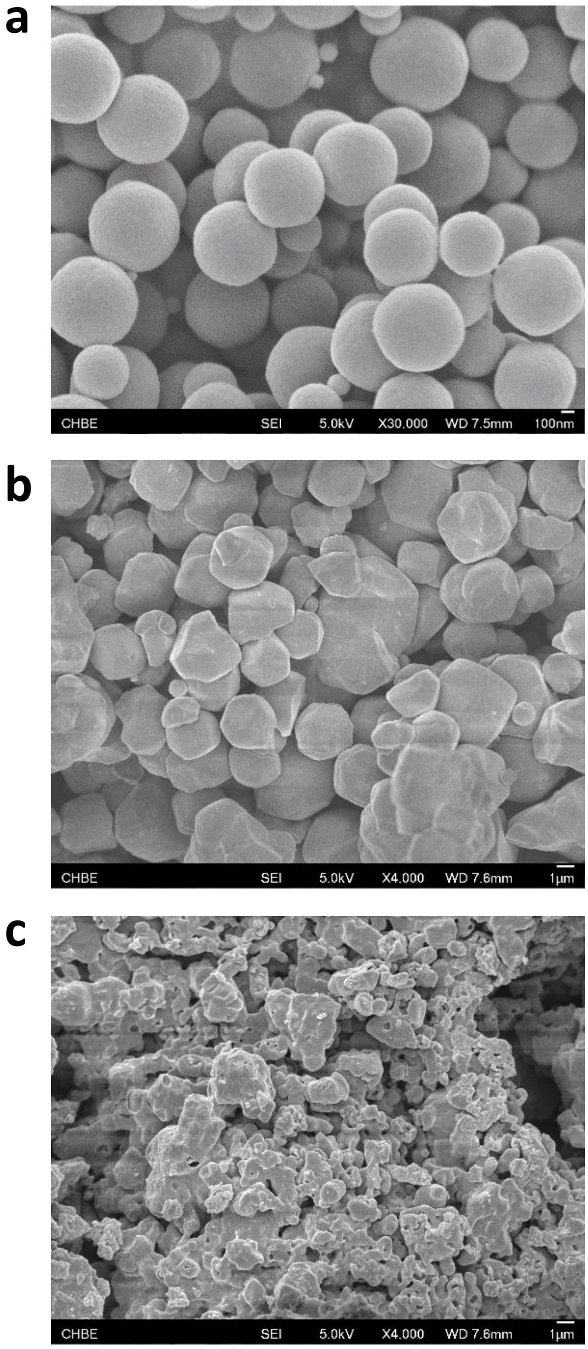

Supplementary Figure 19 | Field emission scanning electron microscopy. FESEM images of **a** 0.2 Rh/NPMA; **b** 0.2 Rh/NSTA and **c** 0.2 Rh/NSMA.

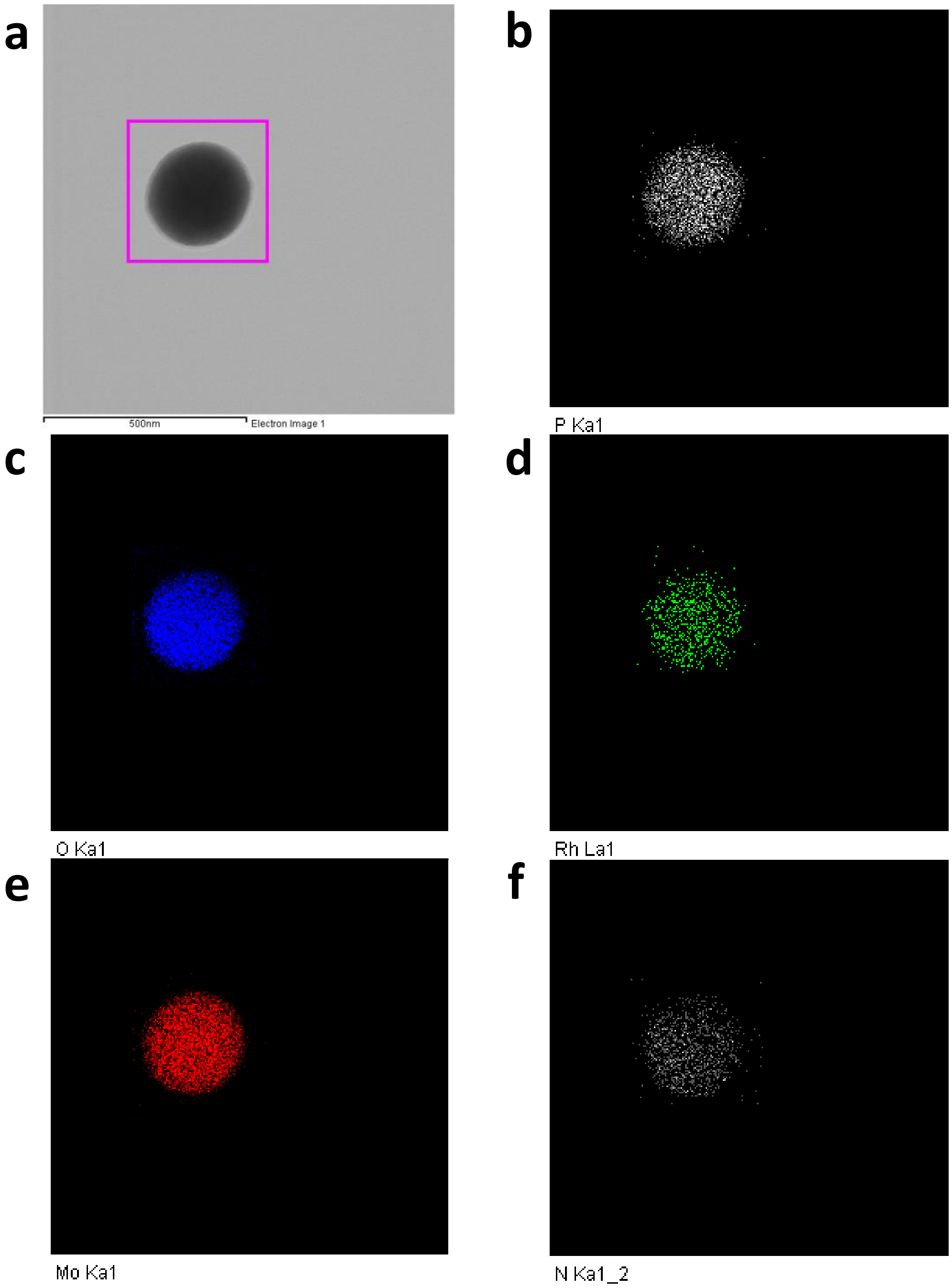

Supplementary Figure 20 | TEM and EDX of 0.2 Rh/NPMA. a TEM image of the 0.2 Rh/NPMA catalyst particle used for EDX analysis. EDX mapping results for **b** P, **c** O, **d** Rh, **e** Mo and **f** N.

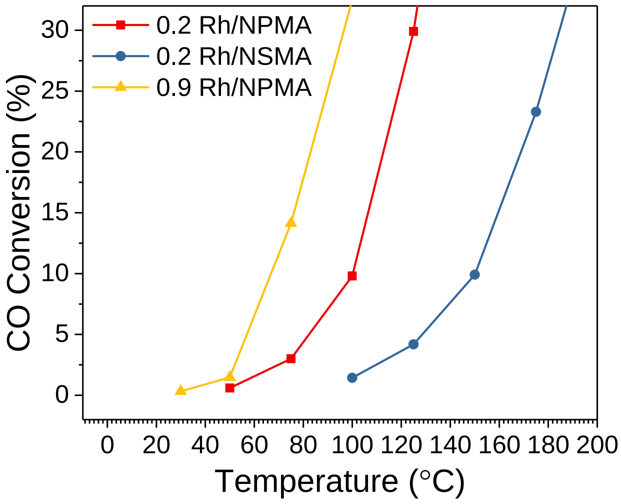

Supplementary Figure 21 | CO oxidation activity. Temperature-activity curves for three different Rh/NHPA, catalyst loading 100 mg, GHSV = 24000 h^{-1} 1% CO/1% O_2, balance Ar.

Supplementary Table 1 | CO vibrational spectra. Predicted and experimental CO vibration spectrum for the structure without oxygen vacancy and 2 adsorbed CO molecules (first two rows) and the structure with oxygen vacancy and 2 adsorbed CO molecules (last two rows). See experimental section for details on the calculations.

	**Predicted wavenumber [cm^{-1}]**	**Predicted intensity [10^{-40} esu^2 cm^2]**	**Experiment [cm^{-1}]**
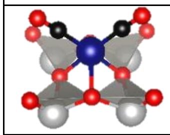	2138	1838	2110
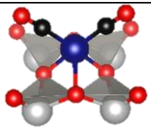	2112	610	2040
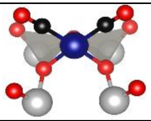	2090	1388	2100
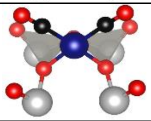	2043	607	2032

Supplementary Table 2 | Reference W 4*f*$_{7/2}$ binding energies. Oxidation state of different tungsten species with their respective W 4*f*$_{7/2}$ binding energies according to previous studies.

Tungsten compound (oxidation state)	W 4*f*$_{7/2}$ Binding energy [eV]	Reference
$W(VI)O_3$	35.6	[2]
$W(VI)O_3$	35.5	[3]
$W(VI)O_3$	35.5	[4]
$H_4PW(VI)_{11}V_1O_{40}$	36.5	[5]
$H_4SiW(VI)_{12}O_{40}$	36.4	[6]
$W(V)O_x$	34.4	[2]
$H_xW(V)O_3$	33.7	[3]
$W(IV)O_2$	32.7	[2]
$W(IV)O_2$	32.5	[3]
$W(IV)O_2$	32.5	[4]
Metallic W	31.2	[2]
Metallic W	31.0	[3]
Metallic W	31.2	[4]

Supplementary References

1 Liu, C.-G., Jiang, M.-X. & Su, Z.-M. Computational Study on M_1/POM Single-Atom Catalysts (M = Cu, Zn, Ag, and Au; POM = $[PW_{12}O_{40}]^{3-}$): Metal–Support Interactions and Catalytic Cycle for Alkene Epoxidation. *Inorg. Chem.* **56**, 10496-10504 (2017).

2 Alov, N. V. Determination of the States of Oxidation of Metals in Thin Oxide Films by X-Ray Photoelectron Spectroscopy. *J. Anal. Chem.* **60**, 431-435 (2005).

3 Fleisch, T. H. & Mains, G. J. An XPS study of the UV reduction and photochromism of MoO_3 and WO_3. *J. Chem. Phys.* **76**, 780-786 (1982).

4 Colton, R. J. & Rabalais, J. W. Electronic structure to tungsten and some of its borides, carbides, nitrides, and oxides by x-ray electron spectroscopy. *Inorg. Chem.* **15**, 236-238 (1976).

5 Venkateswara Rao, K. T., Haribabu, B., Sai Prasad, P. S. & Lingaiah, N. Vapor-phase selective aerobic oxidation of benzylamine to dibenzylimine over silica-supported vanadium-substituted tungstophosphoric acid catalyst. *Green Chem.* **15**, 837-846 (2013).

6 Dziembaj, R., Małecka, A., Piwowarska, Z. & Bielański, A. XPS study of polyaniline supported dodecatungstosilicic acid catalyst. *J. Mol. Catal. A: Chem.* **112**, 423-430 (1996).
