# Supplementary material for: In situ spectroscopy-guided engineering of rhodium single-atom catalysts for CO oxidation
Source: Nat Commun. 2019 Mar 22;10:1330. doi: 10.1038/s41467-019-09188-9 (PMC6430772; doi:10.1038/s41467-019-09188-9)
Supplement: Supplementary file 2 — Peer Review [file 41467_2019_9188_MOESM2_ESM.pdf]

Reviewers' comments:

Reviewer #1 (Remarks to the Author):

The authors present a combined in situ XAS, XPS, DRIFTS and DFT study on single atom Rh catalysis supported on phosphotungstates for the C) oxidation reaction. The main result of the this study is a clear delineation of the mechanistic pathways for this reaction at summarized in Figure 5d. The unique aspect of this mechanistic work is that the authors are abe to track the changes in the charge distribution as Rh and W center change charge state, as well as vibration signature in the presence of CO, O2 and CO/O2. The work is comprehensive and interesting and in principle could be a good addition to Nat. Comm. On the other hand there are a few places where I think the discussion could be more clear.

1, It would clarify the discussion if the authors could write out their mechanism in terms of chemical reactions ex a◊b b◊c to simplify the presentation. The explicit discussion of the mechanism comes at the end of the paper in figure 5d which is not very clear. I do believe their evidence supports most of their proposed mechanism but this is the most critical part of the paper and it needs to come out more. How unique is this mechanism compared to other propositions in the literature?

2, The DFT theory seems to be a missed opportunity-it si underutilized. I would ask does the DFT structural models adequately reproduce the DRIFTS data-vibrational analysis is a standard tool. How does DFT corroborate the reaction energetics-are the proposed intermediates consistent with neither being too high/low w.r.t. free energies from each other. This could in principle be a nice complement to the experimental data set.

In total, I like this paper but I believe it didoes require a bit more work to be fully convincing.

Reviewer #2 (Remarks to the Author):

The authors have performed a detailed characterization of a Rh single atom catalysts on phosphotungtic acid. The authors have done a careful study of the nature of the Rh species when treated in CO, O2 and in the O2/CO mixture. They used EXAFS to infer the oxidation state of the Rh which was corroborated via XPS. DRIFTS measurements revealed the nature of adsorbed CO on the catalyst.

The work builds on their previous publication (ref 47) providing additional detail. But the new insights are limited. They had proposed a very similar reaction mechanism in this previous reference, and the individual reaction steps were elucidated using a very similar approach. Hence, I am concerned about the novelty of the present communication vis a vis ref 47. For instance, they also reported in ref 47 that oxidation of the Rh required higher temperatures than the reduction by CO.

I am also concerned that these catalysts are not very active for this reaction, since the onset temperature is quite high (423K). Hence they do not represent the state of the art in CO oxidation over Rh SACs. For example, a recent manuscript (DOI: 10.1021/jacs.8b04613 J. Am. Chem. Soc. 2018, 140, 9558−9565) suggests that that Rh SACs on ceria have onset of CO oxidation reactivity at room temperature. It is clear that the support must play a role.

In summary, the work is carefully done, but provides limited insights into the reaction mechanism during CO oxidation on the more active Rh catalysts. For the catalysts of higher reactivity, it is the creation of oxygen vacancies and not the refilling of oxygen vacancies which is rate limiting. Furthermore, I am not sure if the phosophotungstic support is robust enough to survive the harsh conditions encountered during treatment of auto exhaust. This make the work of limited utility for

understanding mechanisms on other SACs or for this catalyst to serve in the commercial application of catalysts for exhaust emissions control.

Reviewer #3 (Remarks to the Author):

This paper reports some in-situ and Operando measurements on a novel CO oxidation catalyst consisting of single Rh ions supported on phosphotungstic acid. The measurements are carried out to a high standard and the information obtained will be of interest to researchers in catalysis. However, I do not think the work is of sufficient breadth to be considered for a general science journal such as nature communications.

There are several points that the authors need address before the manuscript could be thought of as suitable for publication.

Firstly, the phosphotungstic acid materials have been used as oxidation catalysts for a number of years with various dopants added. It seems difficult to me to distinguish the current SAC material in which a Rh cation is stabilised on these structures from a traditional doped phosphotungstic acid. Secondly the paper uses some nice in-situ and operando techniques which are said to be good structural probes in this case due to the uniformity of the material. But as oxygen is drawn from the support in the reaction the environment of Rh will change during the reaction so that at any stage Rh atoms will be in different environments and the probes used here will then provide the average of those active sites that have been reduced and those that have not.

Reference to gas compositions are given but not the actual pressure used in each experiment. For example in Figure 1 caption the phrase “different atmospheres” does not give the reader the actual pressure of the gas used. This is particularly important when results from techniques are compared. It is unlikely that the same gas partial pressures are used throughout.

On a minor level the authors should check that the graph shown in figure 1a is really the intended figure. It looks like the experimental procedure diagram rather than the XAS data.

Reviewer #1 (Remarks to the Author):

The authors present a combined in situ XAS, XPS, DRIFTS and DFT study on single atom Rh catalysis supported on phosphotungstates for the C) oxidation reaction. The main result of the this study is a clear delineation of the mechanistic pathways for this reaction at summarized in Figure 5d. The unique aspect of this mechanistic work is that the authors are able to track the changes in the charge distribution as Rh and W center change charge state, as well as vibration signature in the presence of CO, O2 and CO/O2. The work is comprehensive and interesting and in principle could be a good addition to Nat. Comm. On the other hand there are a few places where I think the discussion could be more clear.

1, It would clarify the discussion if the authors could write out their mechanism in terms of chemical reactions ex a b b c to simplify the presentation. The explicit discussion of the mechanism comes at the end of the paper in figure 5d which is not very clear. I do believe their evidence supports most of their proposed mechanism but this is the most critical part of the paper and it needs to come out more. How unique is this mechanism compared to other propositions in the literature?

Response:

Thanks for the valuable feedback. On multiple occasions in the revised MS, we have added simplified chemical reaction equations and short discussions to further highlight and explain the key findings (Eq 1-4, on pages 10, 11 and 13).

In previous reports, all three major types of reaction mechanisms (Langmuir-Hinshelwood, Eley-Rideal and Mars-van-Krevelen) are reported and the vast majority of mechanistic understanding relies on DFT calculations. Although single-atom catalyst systems on reducible supports seem to mostly follow an MvK mechanism, the rate-determining steps (based on kinetic experiments, DFT or both) are often unknown. In the cases where the rate-limiting step was known, the reaction was proposed to be limited mostly by the reaction between CO and the lattice oxygen atom (*Science* **2017**, 358, 1419–1423), the oxidation of the second CO molecule (*J. Phys. Chem. C* **2017**, 121, 11281−11289) or the support cleavage of dioxygen on the surface (*Mol. Catal.* **2019**, 462, 37-45). To the best of our knowledge, a reaction order of 1 towards O_2 combined with 0 for CO has not been shown for SACs before. This is the main uniqueness of our finding.

2, The DFT theory seems to be a missed opportunity-it si underutilized. I would ask does the DFT structural models adequately reproduce the DRIFTS data-vibrational analysis is a standard tool. How does DFT

corroborate the reaction energetics-are the proposed intermediates consistent with neither being too high/low w.r.t. free energies from each other. This could in principle be a nice complement to the experimental data set.

Response:

As suggested, we have employed a frequency analysis for the structures optimized for *ab-initio* XANES fittings on the same level of theory as we used throughout the whole study. We used a scaling factor of 0.972 for all calculations as suggested earlier (*Chem. Phys. Lett.* **2015**, 640, 175-179) for this combination of method (M06L) and basis set (LANL2DZ). As expected, two vibrations are predicted that differ in intensity in a similar extent as our experiments (the vibration at higher wavenumbers shows a higher intensity). The exact values are 2138 cm^{-1} for the structure without oxygen vacancy and 2090 cm^{-1} for the oxygen vacancy structure compared to the experimental 2110 and 2100 cm^{-1}. Although certain differences between experiment and theory are observed, they are well within the range of errors normally encountered for vibrational predictions (*J. Phys. Chem. B* **2005**, 109, 18418–18426) on transition metal-adsorbed CO. Even predictions on structurally much simpler mass-selected metal carbonyl species show errors of up to 50 cm^{-1} (*J. Am. Soc. Mass Spectrom.* **2010**, 21, 739–749).

As such, the general tendency of the vibrational shift towards lower wavenumbers upon reduction of our catalyst (oxygen vacancy formation) is reproduced by DFT predictions. We have added Table S 1 and a section in the main text (page 16) and experimental section relating to CO vibration prediction.

In total, I like this paper but I believe it didoes require a bit more work to be fully convincing.

Response:

Thanks for the positive comments! We hope the revised paper has addressed all reviewer’s comments.

In addition, we have extended the mechanistic study by using the obtained information to design enhanced rhodium single-atom CO oxidation catalysts. By varying the composition of the heteropoly acid supports, the electrochemically determined oxidation potential (in our understanding the energy required to reoxidize the support oxygen vacancy) can be modified easily. More active Rh single-atom catalysts are identified, which substantiate the mechanistic insights from *in situ* spectroscopic study (pages 16-19)

Reviewer #2 (Remarks to the Author):

The authors have performed a detailed characterization of a Rh single atom catalysts on phosphotungtic acid. The authors have done a careful study of the nature of the Rh species when treated in CO, O2 and in the O2/CO mixture. They used EXAFS to infer the oxidation state of the Rh which was corroborated via XPS. DRIFTS measurements revealed the nature of adsorbed CO on the catalyst.

The work builds on their previous publication (ref 47) providing additional detail. But the new insights are limited. They had proposed a very similar reaction mechanism in this previous reference, and the individual reaction steps were elucidated using a very similar approach. Hence, I am concerned about the novelty of the present communication vis a vis ref 47. For instance, they also reported in ref 47 that oxidation of the Rh required higher temperatures than the reduction by CO.

Response:

Thanks a lot for the constructive criticism. Indeed, we have proposed a similar mechanism in the mentioned previous publication. Nevertheless, that proposed mechanism was not based on direct experimental observation, and lacked structural and electronic features of specific intermediates that we are able to show in this work. Direct observations into the changes of all three key components in CO oxidation, i.e., the metal center, the support and the substrate, may not have been demonstrated in a similar manner before.

To enhance the novelty of the paper, and to highlight the predictive ability of the proposed mechanism, we have added very recent results of three new Rh SACs with similar structure compared with the reported Rh_1/NPTA but improved property. Since *in situ* study suggests reoxidation of the support is the rate-determining step, supports with lower oxidation potential should offer more active catalysts. To verify this, we selected silicotungstic acid (STA), bearing an identical oxidation potential with phosphotungstic acid (PTA), as well as silicomolybdic acid (SMA) and phosphomolybdic acid (PMA), which have much lower oxidation potential than STA, as supports to prepare three new Rh_1 SACs. The activity of the new catalysts follow exactly the predictions by the mechanism, and the best Rh_1/NPMA catalyst exhibited a T_{20} value 130 °C below the previously reported Rh_1/NPTA (drop from 256 °C to 112 °C). It is still less active than the best Rh SAC reported in the literature (*J. Am. Chem. Soc.* **2018**, 140, 9558–9565), but it highlights the usefulness of the mechanism in guiding new catalyst design.

I am also concerned that these catalysts are not very active for this reaction, since the onset temperature is quite high (423K). Hence they do not represent the state of the art in CO oxidation over Rh SACs. For example, a recent manuscript (DOI: 10.1021/jacs.8b04613 J. Am. Chem. Soc. 2018, 140, 9558−9565) suggests that that Rh SACs on ceria have onset of CO oxidation reactivity at room temperature. It is clear that the support must play a role.

Response:

Thank you for the feedback. True, the CO oxidation activity for the Rh_1/NPTA catalyst is lower than what has been shown previously for other metals and rhodium. Our intention was to select a reliable, structure-well known catalyst for mechanistic study, hoping that the knowledge and insights generated become useful in designing new catalysts.

Indeed, the mechanism helped us come up with catalysts with significantly improved CO oxidation performance (also see response to the last comment). As expected from the reaction mechanism, a support that is easier to be reoxidized should enhances the CO oxidation activity. The easy tunability of the redox properties of the heteropoly acids render them particularly useful for the elucidation of the support effect on the single-atom catalyzed CO oxidation. A newly developed Rh_1/NPMA catalyst exhibit catalytic onset slightly higher than room temperature and is approaching the activities reported for the state-of-the-art SACs. We do acknowledge that the estimated TOFs are still lower than those presented in the above-mentioned JACS paper even with our enhanced catalysts, but the activity may be further improved in the future given the availability and tunability of heteropoly acids with various structures and redox properties.

In summary, the work is carefully done, but provides limited insights into the reaction mechanism during CO oxidation on the more active Rh catalysts. For the catalysts of higher reactivity, it is the creation of oxygen vacancies and not the refilling of oxygen vacancies which is rate limiting.

Response:

Thanks for the very valuable feedback. In the revised MS, we have included new Rh SACs supported on heteropoly acids with comparable activity to most previous studies. For this entire Rh catalyst series, the activity correlates linearly with the oxidation potential of the heteropoly acid support indicating that the support reoxidation is still the rate-determining step. In agreement with this is the *in situ* DRIFT study of the best new catalyst (Rh_1/NPMA) which is reduced at room temperature but is reoxidized at a higher temperature.

There are heteropoly acids where the oxidation potential was reported to be negative by a few hundred mV (based on electrochemical measurements) and we believe that in those cases the CO oxidation reaction should occur at even lower temperatures. We agree with the reviewer that the reoxidation of the oxygen vacancy might not be rate determining anymore for these catalysts, and we are currently planning and executing syntheses and experiments along this direction.

Furthermore, I am not sure if the phosophotungstic support is robust enough to survive the harsh conditions encountered during treatment of auto exhaust. This make the work of limited utility for understanding mechanisms on other SACs or for this catalyst to serve in the commercial application of catalysts for exhaust emissions control.

Response:

This is another excellent point. We have not investigated the CO oxidation performance of our catalysts under more realistic simulated auto exhaust conditions. Attempting the oxidation of CO in hydrocarbon-containing atmospheres would certainly be interesting. Given that we have extended the manuscript along the direction of understanding the CO oxidation mechanism and predicting the design of enhanced catalysts for lower temperature application, we feel that going along this direction may be beyond the scope of this publication.

Considering the high-temperature stability, Rh_1/NPTA catalyst can be recycled multiple times after CO oxidation at up to 400 °C and that the catalyst materials are stable up to 500 °C based on TGA analysis reported in our previous study. We have also demonstrated in the revised manuscript by means of IR and Raman spectroscopy that the heteropoly acid structure are fully maintained after CO oxidation reaction at 300 °C (Figure 5h, and Figure S 17). Although we understand that temperature conditions in automotive exhausts exceed the above-mentioned values, the high-temperature performance and stability of our catalysts are on par with most previously reported single-atom catalyst systems for CO oxidation.

Reviewer #3 (Remarks to the Author):

This paper reports some in-situ and Operando measurements on a novel CO oxidation catalyst consisting of single Rh ions supported on phosphotungstic acid. The measurements are carried out to a high standard and the information obtained will be of interest to researchers in catalysis. However, I do not think the work is of sufficient breadth to be considered for a general science journal such as nature communications.

Response:

Thank you for sharing your reasonable concerns. We agree that a pure *in situ* spectroscopic study even of a very relevant single-atom catalyst system might not suffice a journal like Nat. Commun. Therefore, we have extended the scope of our manuscript by using the key findings to develop new catalysts that are significantly more active than the initially investigated one. Since *in situ* study suggests reoxidation of the support is the rate-determining step, supports with lower oxidation potential should offer more active catalysts. Using this principle, several new Rh catalysts supported on heteropoly acids bearing lower oxidation potential were developed. The best in the series exhibited a T_{20} value (temperature at which 20% CO conversion is achieved) 130 °C below the catalyst originally used in *in situ* study, and is approaching the state-of-the-art Rh catalyst for CO oxidation in the literature.

In the revised MS, we have thus added an additional section to the manuscript and one more figure (Figure 5 after combining Figure 1 and 2 into a single figure) to describe how the mechanistic insights lead us to the discovery of more active catalysts for CO oxidation. Also we have changed the title as “In situ Spectroscopy-Guided Engineering of Rhodium Single-Atom Catalysts for CO Oxidation” since the manuscript does not only include mechanistic study, but also mechanism-assisted new catalyst development. Short sections are also added to the abstract, introduction and conclusion, and 6 related figures are added to the supporting information.

In this way, the manuscript with its predictive ability in designing new catalytic materials is now more broadly relevant to a readership of materials-, catalysis-, spectroscopy- or organometallics-oriented researchers and beyond.

There are several points that the authors need address before the manuscript could be thought of as suitable for publication.

Firstly, the phosphotungstic acid materials have been used as oxidation catalysts for a number of years with various dopants added. It seems difficult to me to distinguish the current SAC material in which a Rh cation is stabilised on these structures from a traditional doped phosphotungstic acid.

Response:

Thanks for the helpful feedback. Although transition metal-doped heteropoly acids have been previously utilized for a range of reactions and in particular oxidation reactions, we are not aware of Rh-doped self-supported heteropoly acids prepared with similarly high weight loadings for CO oxidation. Furthermore, a deep understanding about the (electronic) structure after synthesis and the structural dynamics during gas-phase reactions were missing for previous metal-doped heteropoly acid salts. The major purpose of our work is not to develop an entirely new catalyst, but to use a structurally well-defined one for detailed mechanistic study.

Secondly the paper uses some nice in-situ and operando techniques which are said to be good structural probes in this case due to the uniformity of the material. But as oxygen is drawn from the support in the reaction the environment of Rh will change during the reaction so that at any stage Rh atoms will be in different environments and the probes used here will then provide the average of those active sites that have been reduced and those that have not.

Response:

The reviewer has raised an important point. Indeed, the structure and local geometry around the metal atoms will change during the reaction or under exposure to reagents. We are aware of this, and mentioned in the manuscript "A major disadvantage of *in situ* spectroscopy study is that for spectra measured at an ensemble level an averaged signal of all responsive species is collected, which may hide the information from the real active species and thus be misleading. Only when all species are identical will the spectra reflect accurate information of individual species, and only then the spectra could be conveniently used to reveal structural information and reaction mechanism."

As pointed out by the reviewer, the uniformity of the material is essential. And that is the major reason we use heteropoly acid supported rhodium single-atom catalyst for the study. Based on our previous work, every charged Rh species in this material locates on structurally well-defined heteropoly acid which further assembles into a mesoporous structure. As such, all Rh species are in principle identical and a vast majority

of rhodium species contributes to the reaction due to the presence of abundant mesopores. The clearly defined coordination environment around the rhodium atoms mean that all rhodium atoms on average experience the same changes during the reaction, or under exposure to a reagent, and thus in principle exhibit the same reactivity and local structure under a given condition.

There are experimental evidence to support this. From both *in situ* CO-DRIFT and XANES spectra, full conversion of Rh^{3+} species to Rh^{1+} species was observed when the catalyst was exposed to CO gas, while Rh species quantitatively changed back to 3+ state in the presence of O_2. In addition, the valent state of Rh is 3+ instead of being a value between 1+ and 3+ when a mixture of CO and O_2 is added. These observations suggest that all Rh species responds to reagents in a similar manner, and give us confidence to assign several structural models to reaction intermediates.

Reference to gas compositions are given but not the actual pressure used in each experiment. For example in Figure 1 caption the phrase "different atmospheres" does not give the reader the actual pressure of the gas used. This is particularly important when results from techniques are compared. It is unlikely that the same gas partial pressures are used throughout.

On a minor level the authors should check that the graph shown in figure 1a is really the intended figure. It looks like the experimental procedure diagram rather than the XAS data.

Response:

Thank you for the valuable feedback. We have added the specific partial pressures of the reactant gases which were essentially the same for the *in situ* DRIFTS, XAS and CO oxidation activity measurements (on page 17, 22, 23 and 25 respectively). Only for *in situ* XPS measurements, we had to rely on partial pressures approximately two orders of magnitude lower (0.5 mbar compared to 25 mbar for most other techniques).

Figure 1a is indeed intended to show the experimental protocol which is very similar for all *in situ* techniques applied throughout the manuscript and is added to remove ambiguity regarding the experimental procedures. The reviewer is right to point out that it looks like an experimental procedure, but it helps the readers to correlate various spectra with the condition under which they are collected. Prompted by the reviewer's comment, we have combined the original figure 1 and 2 into the new figure 1 so that the figure carries more compact information, and it facilitates readers to correlate experimental condition with corresponding spectra.

All revisions are highlighted in yellow in the main text.

For reviewers' convenience, we attach below the new section (the most important addition in the revised MS) that has been added.

Engineering more efficient CO oxidation catalysts

From above, the reoxidation of the HPA support is the rate-limiting step during the CO oxidation. Designing a catalyst that has a lower barrier for the reoxidation should thus enhance the activity significantly. HPAs have been shown to be redox-tuneable by changing the central atom of the whole Keggin unit (P or Si) or the central atom of the MO_4 subunits (M = Mo, W) (Figure 5a). To ensure full atomic dispersion of rhodium for all SACs based on those HPAs we synthesised them following the same procedure as reported previously with a weight loading of 0.2%. ATR-IR (Figure S 16), Raman spectroscopy (Figure S 17) and XRD (Figure S 18) confirms the intact structure of the heteropoly acids after adsorption of rhodium and precipitation as ammonium salt. We also confirmed the atomic dispersion of rhodium on the new catalysts by XAS and CO DRIFTS. CO DRIFTS analysis of the Rh/NPMA catalyst showed the occurrence of two peaks at 2104 and 2034 cm^{-1}. Compared to rhodium on NPTA, the CO absorption wavelengths are slightly shifted towards lower wavenumbers, and that the rhodium supported on NPMA is reduced at slightly decreased temperatures than rhodium on NPTA (300 K vs. 323 K, Figure 5b). For 0.2 Rh/NSTA and 0.2 Rh/NSMA, two peaks are observed with positions of the symmetric vibration peaks at 2105 for NSTA and 2097 cm^{-1} for NSMA, respectively, after a reductive pretreatment (Figure 5c and 5d), suggesting the single-atom identity. Similar to the initially studied PTA-based catalyst, we observed a high whiteline intensity very close to the Rh_2O_3 sample and the absence of significant scattering contributions from shells above the first Rh–O contribution for 0.2 Rh/NSTA (Figure 5e and 5f). The strong X-ray absorption by molybdenum close to the Rh X-ray edge prevented the XAS analysis for the two molybdenum-based samples. SEM analysis reveals that the morphology of the Rh_1/NPMA catalyst is almost the same as for Rh_1/NPTA with an EDX pattern revealing

homogeneous distribution of Rh, Mo, P, N and O whereas 0.2 Rh/NSTA and 0.2 Rh/NSMA exhibit a slightly different morphology (Figure S 19 and S 20).

CO oxidation reactions were performed in a temperature range of 50-300 °C (323-573 K) with a GHSV of 24000 h^{-1} and a partial pressure of each 0.01 bar for CO and O_2. Distinct differences in the temperature behaviour were observed, with the two Mo-based ones showing significant activity even at around 50 °C (323 K). The T_{20} values (temperature at which 20% CO conversion is achieved) of for NPMA and NSMA are 112 °C (385 K) and 169 °C (442 K) respectively. In comparison, the tungsten-based catalysts only exhibit activity at higher temperatures of 256 °C (529 K) for silicotungstic acid (NSTA) and 244 °C (517 K) for NPTA (Figure 5e). ATR-IR and Raman spectroscopy show that even after CO oxidation reaction at temperatures up to 300 °C (573 K), the heteropoly acid structure remains intact (Figure 5h) and no formation of metal oxides were observed. In situ DRIFTS studies reveal that the reoxidation of reduced rhodium atoms on PMA occurs after being exposed to O_2 at temperatures as low as 323 K for around 5 min indicated by a shift of the CO vibration from 2096 cm^{-1} back to 2105 cm^{-1} (Figure 5i). The activation energies for 0.2 Rh/NPTA and 0.2 Rh/NSTA are very similar at 126 and 121 kJ/mol respectively whereas 0.2 Rh/NPMA exhibits a significantly lower activation energy of 55 kJ/mol in accordance with the significantly higher CO oxidation activity (Figure 5j). A CO oxidation reaction test with 0.9 Rh/NPMA reveals an even lower T_{20} value (82 °C, 355 K) as compared to its lower weight loading counterpart (Figure S 21).

Although it proves difficult to experimentally determine the redox potential of solid catalysts, oxidation potentials of various HPAs have been determined based on electrochemical measurements in solution[65]. We found that the T_{20} values for CO oxidation indeed correlates very well with the oxidation potentials (Figure 5k) proving the predictive power derived from the mechanistic understanding of CO oxidation SACs, i.e., the redox tunability of HPAs can be used synergistically to engineer enhanced CO oxidation catalysts.

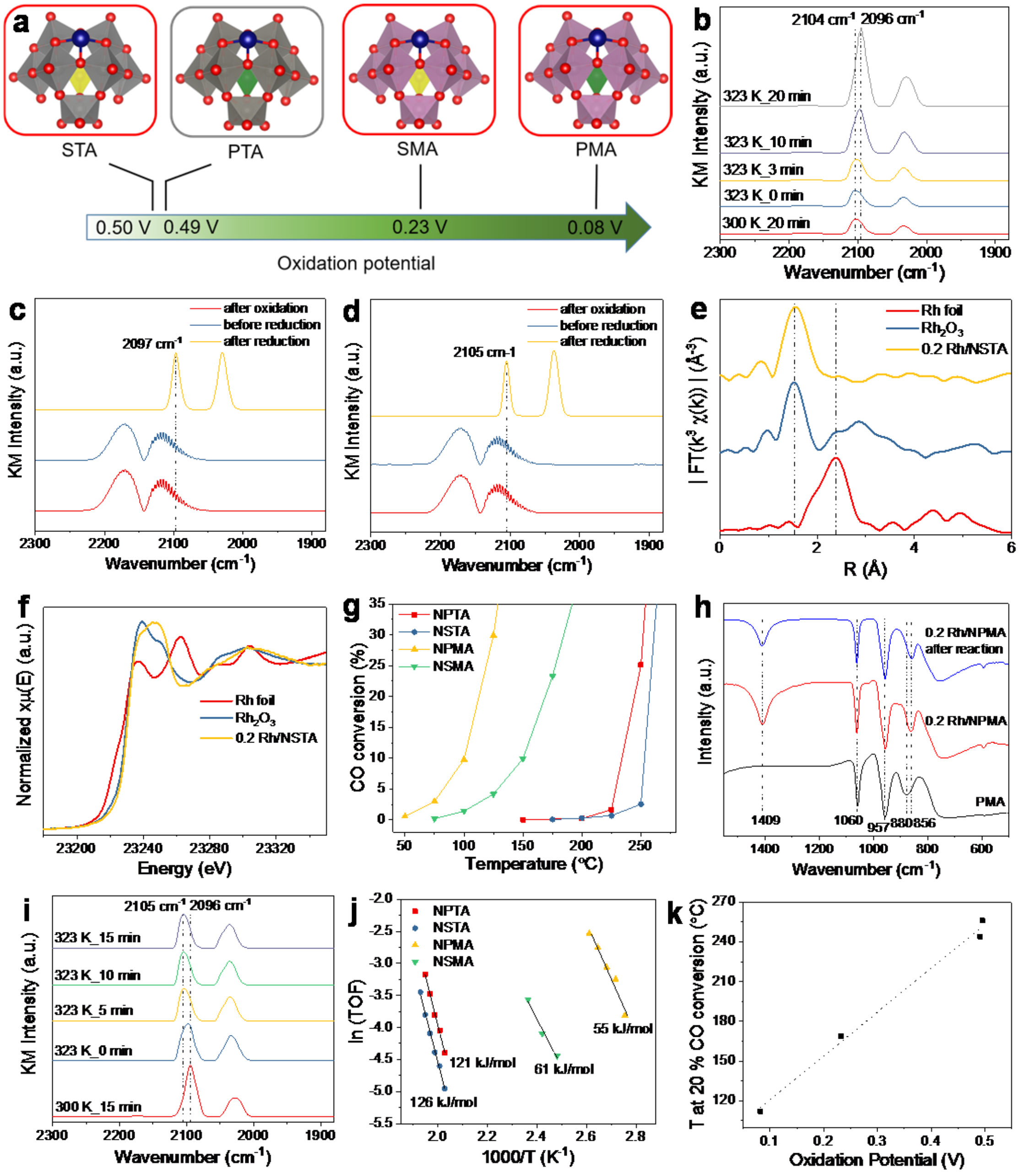

Figure 1. Design principle, *ex*/*in situ* characterization and catalytic performance of different 0.2 Rh/NHPA catalysts. **a** Oxidation potentials of 4 HPAs (grey indicates the initially studied and red the newly synthesized catalysts); CO DRIFT for **b** 0.2 Rh/NPMA, **c** 0.2 Rh/NSMA and **d** 0.2 Rh/NSTA, oxidation for **c** & **d** was carried out at 523 K for 1 h under 5% O_2 and reduction was performed using 5% CO for 30 min at 373 K; **e** k^3-weighted EXAFS spectra and **f** XANES for rhodium foil, Rh_2O_3 and 0.2 Rh/NSTA; **g** Temperature-activity curves for Rh catalysts. Catalyst loading 100 mg, GHSV = 24000 h^{-1} 1% CO/1% O_2, balance Ar; **h** ATR-IR spectra for PMA, 0.2 Rh/NPMA before and after CO oxidation reaction up to 300 °C (573 K); **i** *in situ* DRIFT spectra for the reoxidation of reduced 0.2 Rh/NPMA under 5% O_2 atmosphere; **j** activation energies of the catalysts for CO oxidation; **k** correlation between T_{20} and oxidation potential of the support.

REVIEWERS' COMMENTS:

Reviewer #1 (Remarks to the Author):

I have read the revised version of the manuscript and the detailed response to the reviewers. Overall, the manuscript has improved significantly and may be appropriate for publication

Reviewer #2 (Remarks to the Author):

The authors have addressed all of the reviewer comments. This is a carefully conducted study of a single atom catalyst, elucidating some of the key steps during CO oxidation. My concern is that the HPA is a very specialized support and not the most beneficial for the CO oxidation reaction. Hence, the conclusions are not very insightful for the reaction being studied, since the rate limiting step for this system is very different from other reducible oxides.

Reviewer #3 (Remarks to the Author):

The authors have now provided a revision of the manuscript following the first round of reviewing. For my part their responses clearly show that they have considered the points raised and made reasonable adjustments to the paper. This has greatly improved the clarity of presentation and I am happy to now recommend publication.

Responses to Reviewers.

Reviewer #1 (Remarks to the Author):

I have read the revised version of the manuscript and the detailed response to the reviewers. Overall, the manuscript has improved significantly and may be appropriate for publication

Response:

Thank you. Your efforts are greatly appreciated.

Reviewer #2 (Remarks to the Author):

The authors have addressed all of the reviewer comments. This is a carefully conducted study of a single atom catalyst, elucidating some of the key steps during CO oxidation. My concern is that the HPA is a very specialized support and not the most beneficial for the CO oxidation reaction. Hence, the conclusions are not very insightful for the reaction being studied, since the rate limiting step for this system is very different from other reducible oxides.

Response:

Thank you. We agree that heteropoly acids represent a rather special support and thus understand your concerns regarding the generality of our findings about Rh_1/NHPA catalysts but we also like to point out four aspects:

1. Our claims mostly are directed towards the increased use of in situ/operando spectroscopy (ideally on similarly well-defined structures) for reasons that are highlighted multiple times throughout our manuscript including the (in principle) contribution of all metal species towards the catalytic reaction. Therefore, findings about the CO oxidation reaction might be of limited use for other catalysts but we believe that the proposed approach holds promise to understand single-atom catalysts better.
2. Almost every finding about single-atom catalyst systems are specific to a certain metal, support material or preparation method and only very few examples provide a robust procedure that combine any of the two above parameters.
3. Although we only report Rh_1 on four different commercially available heteropoly acids with Keggin structure, hundreds of more heteropoly acids are reported with many different structures. Therefore, they represent a very versatile support that can be extended to participate in a range of other catalytic reactions.
4. Heteropoly acids offer us the opportunity to engineer our support in a way that allows us to tune the redox properties for enhancing the CO oxidation activity. There seems to be no reported support system that allows us to follow a similar approach for changing the metal support interaction.

Reviewer #3 (Remarks to the Author):

The authors have now provided a revision of the manuscript following the first round of reviewing. For my part their responses clearly show that they have considered the points raised and made reasonable adjustments to the paper. This has greatly improved the clarity of presentation and I am happy to now recommend publication.

Response:

Thank you. Your efforts are greatly appreciated.
